# Exploring Genetic Therapies Targeting Amyotrophic Lateral Sclerosis in Animal Models: A Systematic Review and Meta‐Analysis

**DOI:** 10.1002/jgm.70106

**Published:** 2026-08-18

**Authors:** Hannah E. Wedgwood, Richard I. Tuxworth, Zubair Ahmed

**Affiliations:** ^1^ Department of Inflammation and Ageing, School of Infection, Inflammation and Immunology University of Birmingham Birmingham UK; ^2^ Department of Cancer and Genomic Sciences, School of Medical Sciences, College of Medicine and Health University of Birmingham Birmingham UK; ^3^ Centre for Neurogenetics University of Birmingham Birmingham UK; ^4^ University Hospitals Birmingham NHS Foundation Trust Birmingham UK

**Keywords:** ALS, amyotrophic lateral sclerosis, genetic therapy, in vivo studies

## Abstract

**Background:**

Amyotrophic lateral sclerosis (ALS) is a rare, neurodegenerative disease, for which there is currently no known cure. ALS primarily affects motor neurons, with rapid deterioration, meaning symptoms develop quickly, from problems with speech and muscle weakness to breathing issues and paralysis. This systematic review aimed to explore the preclinical efficacy of various genetic therapies used to target ALS using in vivo rodent models.

**Methods:**

In vivo studies of genetic therapies targeting ALS and its symptoms published between January 2015 and December 2025 were included in this review. The following databases were used: Web of Science, Scopus and PubMed. The primary outcome investigated was the total number of motor neurons, with secondary outcomes of rodent survival and muscle function by observing rotarod performance also being analysed. The SYRCLE tool was used to assess risk of bias in included studies.

**Results:**

Of the 451 studies identified by searching the databases, 53 studies were found to be eligible for this systematic review. The articles were divided into subcategories depending on the gene target of each therapy. Meta‐analysis of outcomes within appropriate studies showed significant improvements for the majority of selected outcomes (*p* < 0.05), favouring genetic therapy intervention.

**Conclusions:**

Results suggest that genetic therapies in rodent models targeting ALS are effective. However, due to a high risk of bias in preclinical studies, further high‐quality studies are warranted to support this conclusion and onward translation into the clinic.

## Graphical abstract

Amyotrophic lateral sclerosis (ALS) is a rare neurodegenerative disease for which there is currently no known cure. This systematic review explores the efficacy of genetic therapies used to target ALS in preclinical studies using in vivo rodent models. Outcomes investigated include the total number of motor neurons, rodent survival, and muscle function by observing rotarod performance. Meta‐analysis of outcomes within appropriate studies showed significant improvements for most selected outcomes (*p* < 0.05), favouring genetic therapy intervention. However, further high‐quality studies are required to support this conclusion and onward translation into the clinic, due to a high risk of bias in preclinical studies.

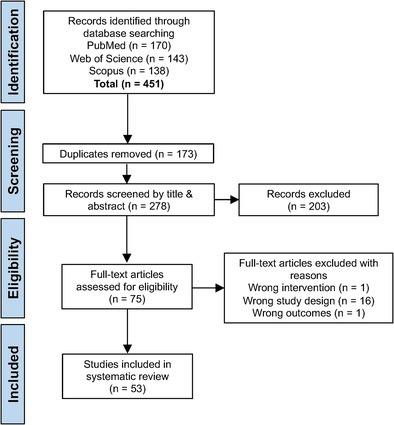

## 1 Introduction

Amyotrophic lateral sclerosis (ALS) is a rare neurodegenerative disease characterised by the progressive degeneration of upper and lower motor neurons (UMNs and LMNs). The disease manifests as muscle function loss, starting as muscle weakness, slurred speech, increased fatigue and problems swallowing. These symptoms typically deteriorate rapidly to complete muscle function loss, respiratory failure and eventually death 2–5 years after clinical onset [1]. The disease usually begins at 50–60 years of age [2], with the exception of some rare cases of juvenile ALS [3]. Both familial and sporadic cases of ALS are seen, with sporadic ALS much more common (90%–95% of cases) [4].

ALS is an extremely complex disease, with multiple underlying molecular mechanisms and more than 40 genes known to be associated with it [5]. Most cases are heterogeneous, making identification and treatment more complicated. The most prevalent familial form is caused by a hexanucleotide (GGGGCC) repeat expansion in the first intron of the *C9orf72* gene [6]. The majority of healthy individuals have up to 11 repeats [7], whereas ALS patients can have hundreds to thousands [8, 9]. Other key ALS‐causing genes include *SOD1*, *TARDBP*, *FUS*, *ERBB4* and *ATXN2* [10]. Superoxide dismutase (*SOD1*) was the first gene identified to be associated with ALS [11] and is the second most common form of ALS worldwide. Many different *SOD1* variants are associated with ALS, with variants identified in 21% of familial ALS and 2.3% of sporadic ALS [12], the most common being *D90A*, *A4V* and *G93A*. In most of the studies reviewed here, the authors use transgenic rodents with the *G93A* variant [13].

Genetic therapy employs nucleic acid‐based therapies to silence mutated genes, supplement genes or edit a genome. As opposed to traditional drugs, genetic therapy aims not only to provide relief for symptoms of the disease but also to treat and eradicate the disease from the patient [14]. There are a variety of methods by which this can be achieved. First, gene augmentation is the addition of, or substitution of, a gene to correct a loss‐of‐function defect. For example, Zolgensma encodes cDNA for the *SMN1* gene that is mutated in children affected by spinal muscular atrophy [15]. Second, pathological gain‐of‐function mutations can be suppressed, by using RNA interference (RNAi) or antisense oligonucleotides (ASOs), for example [16]. For ALS, mutations in the *SOD1* gene often cause misfolding and aggregation of the SOD1 protein [17]. Suppressing this mutated gene through genetic therapy could potentially eliminate the symptoms. Examples of approved therapies using these methods include Patisiran, an RNAi therapy to treat a rare disease called hereditary ATTR (hATTR) amyloidosis [18], and Mipomersen, an ASO therapy used to manage Homozygous Familial Hypercholesterolemia (HoFH) [19]. Finally, genetic therapy can be used as a treatment via genome editing by correcting disease‐causing mutations [20]. This approach is used in the approved therapy, Casgevy, which is used to treat sickle cell disease [21].

Another important aspect of genetic therapy is the vector that is being used to deliver the genetic material into the patient. Viral vectors are more commonly used since they have a very high transduction efficiency and can target a large variety of tissues [22]. Commonly used viral vectors include lentiviruses (LVs) and adeno‐associated viral (AAV) vectors [23]. LVs are known to be able to target a range of different tissues and cell types, including the central nervous system. They are particularly useful for ex vivo gene correction but there is a risk of insertional mutagenesis during clinical applications, as well as challenges found in immune‐mediated rejection [24]. AAVs are widely used as vehicles for gene delivery since there are a large range of serotypes of AAV, all of which have varying limitations and benefits. For neurodegenerative diseases, such as ALS, AAV9 is a favoured serotype due to its ability to cross the blood–brain barrier (BBB) and bias for transduction of neurons [25, 26]. Other studies have found that adding a peptide, PhP. B, to AAV9 increased the transduction across the BBB by 40‐fold [27].

Treatments for ALS aim to slow disease progression and extend survival. Three FDA‐approved therapies are currently available: Riluzole, approved in 1994, which reduces neurotoxicity by enhancing glutamate uptake [28] and inhibiting calcium‐dependent release of glutamate [29]; Edaravone, an antioxidant, first approved in 2001 for treatment of acute cerebral infarction [30], which protects neurons by reducing oxidative stress and slowing functional decline; and the most recent, Sodium Phenylbutyrate–Taurursodiol, which decreases neuronal death by reducing endoplasmic reticulum and mitochondrial stress and was approved for treatment in 2022 [31]. However, the goal of these approved therapies is to simply manage symptoms, and so, it has limited efficacy.

Genetic therapies, in contrast, would be more effective at slowing or preventing disease progression. Genetic therapy is a continuously expanding field, and there is hope that genetic therapies may prove effective for ALS. However, different approaches targeting different ALS‐associated genes and tested in a variety of preclinical models have made it difficult to assess how effective genetic therapy for ALS might be. By looking across multiple studies, using various strategies and models, this systematic review and meta‐analysis aimed to determine how effective genetic therapies are in preclinical models of ALS, focussing on motor neuron survival, motor function and overall survival. The outcome of this review highlights promising genetic targets that may ameliorate ALS symptoms, as well as highlight future directions.

## 2 Methods

### 2.1 Search Strategy

The systematic review was conducted to identify studies investigating genetic therapy approaches in ALS, according to the Preferred Reporting of Items for Systematic Reviews and Meta‐analysis (PRISMA) guidelines [32]. The systematic review was also prospectively registered with PROSPERO (CRD420251267973). The literature search was completed on 9 December 2025 across three databases, including PubMed, Scopus and Web of Science. The search targeted publications released between January 2015 and December 2025. Boolean operators OR and AND were utilised to refine the search. For search terms, ‘Amyotrophic Lateral Sclerosis’ OR ‘ALS’ AND ‘gene therapy’ OR ‘genetic therapy’ were used. The search was conducted by HEW and confirmed by a second author, ZA.

### 2.2 Inclusion and Exclusion Criteria

Inclusion criteria for this study consisted of full‐text articles in English, published between January 2015 and December 2025. Eligible studies involved a genetic therapy, focused on targeting ALS in rodent models. Initial exclusion criteria for this review included studies that were not available in English or outside of the January 2015 to December 2025 time frame. Additionally, studies that focused on nongenetic therapy as a therapeutic approach or addressed methodology instead of therapeutic effect of the genetic therapy were excluded.

Four hundred fifty‐one studies were identified from the initial search over the three databases. Following the removal of duplicates and screening titles and abstracts for relevance, 75 studies remained to have their full‐text reviewed. Covidence software helped identify duplicates and follow the screening process [33]. From those 75 publications, 53 of the studies met the inclusion and exclusion criteria, after appraisal by the two reviewers independently.

### 2.3 Data Extraction

Numerical data related to each outcome were extracted from studies that reported this, including *n* numbers, mean and standard deviation (SD) or standard error of the mean (SE) by the primary author (HEW) to a spreadsheet for further analysis, which was then reviewed by the second author (ZA) for verification. Where data were presented as SE, SD was calculated using the following equation: SD = SE × √*n*. Each study was also evaluated for the characteristics of the study, characteristics of the animals involved in the study and characteristics of the intervention and controls. Authorship and origin of the study were included for characteristics of the study. The sample size, sex and animal strains were included for characteristics of the animals involved. Finally, age at injection, delivery method, dosages and controls were included for characteristics of the intervention. Additionally, details for the targets of the genetic therapy, motor neuron survival, motor function and overall survival of animals with and without intervention were collected. Studies that did not contain numerical data were assessed thematically and discussed in a narrative form.

### 2.4 Risk of Bias

The risk of bias was assessed using the SYRCLE's risk of bias tool, which documents 10 domains and has been specifically developed for animal studies [34]. These domains for risk of bias includes sequence generation, baseline characteristics, allocation concealment, random housing, blinding operation, random outcome assessment, blinding outcome assessment, incomplete outcome data, selective outcome reporting and other sources of bias. Two reviewers (HEW and ZA) screened the studies independently and rated each domain for low, unclear or high risk of bias across each study. Any discrepancies between the assessors were discussed to reach a mutual agreement.

### 2.5 Statistical Analysis

For studies that reported numerical data related to each outcome assessed, the *n* number, mean and SD (for studies that presented SEM, SD was calculated) was extracted and used to conduct meta‐analysis using RevMan 4.0 software (Cochrane Collaboration, London, UK), employing a random‐effects model and reporting mean differences and 95% confidence intervals (CI). Heterogeneity was assessed using *I* ^2^, *χ* ^2^ and *τ* ^2^ scores and forest plots were created for each meta‐analysis performed. The forest plots displayed whether the results favoured intervention with the genetic therapy or the control groups. Studies that reported median values for any outcome were excluded from the meta‐analysis because converting medians to means is not mathematically possible without the full dataset, which the authors did not provide or share upon requesting the full dataset from corresponding authors, within a reasonable time.

## 3 Results

This systematic review was conducted according to the PRISMA guidelines [32] and the accompanying completed PRISMA checklist is included in the Supporting Information. No deviations from the originally registered PROSPERO registration record were made during the review process.

### 3.1 Search Results

**FIGURE 1:**
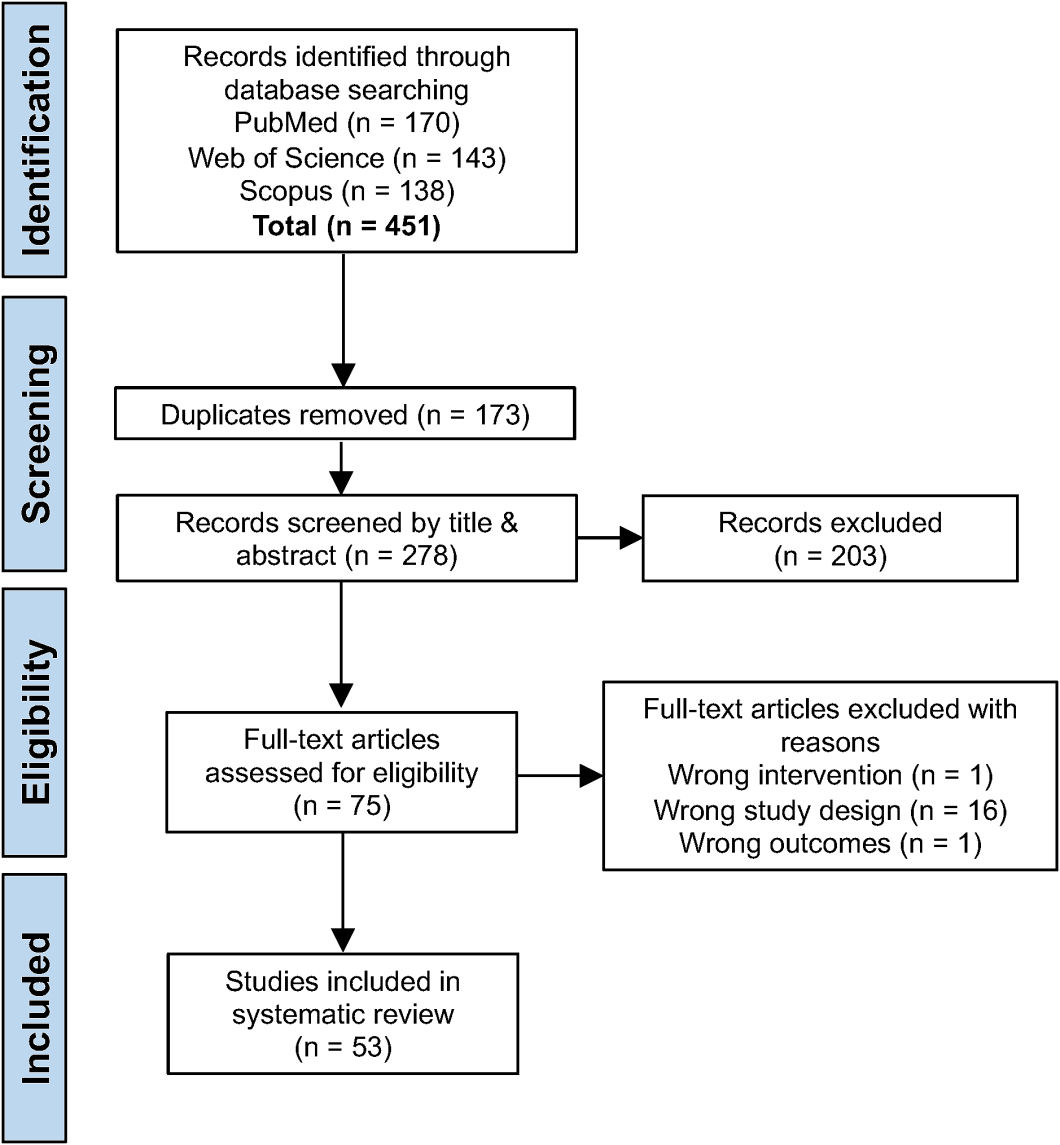
Flow chart showing study selection, following the PRISMA method. The number of studies is shown throughout each stage of the process, along with the relevant exclusion criteria.

The PRISMA flow chart is presented as Figure 1. We identified 451 studies using the inclusion and exclusion criteria, and no further studies were identified through citation searching. After removal of 173 duplicates, 278 studies were screened by title and abstract. From this, 203 studies were excluded, leaving 75 studies eligible for full‐text reading. Eventually, 53 studies remained, and the rest of the studies were excluded for a variety of reasons including wrong intervention, wrong study design and wrong outcomes. For example, a study may not have used a genetic therapy, or has not assessed the primary and secondary outcomes or did not include a comparator group.

### 3.2 Study Characteristics

The characteristics of the included studies have been detailed in Table 1, including study origin, sample size, sex of animal model used, in vivo model, age, dose and method of delivery and controls. The largest share of the studies originated from the United States [35, 36, 37, 38, 39, 40, 41, 42, 43, 44, 45, 46, 47, 48, 49, 50, 51, 52, 53, 68] (24 studies), with the rest spanning 15 countries in total: China [54, 55, 56, 57, 58, 59, 60] (eight studies), Spain [61, 62, 63, 64] (four studies), Japan (four studies), United Kingdom [42, 65, 66] (three studies), France [67, 69] (two studies), Switzerland [67, 70] (two studies), South Korea [48, 71] (two studies), Italy [72, 73] (two studies), Israel [74, 75] (two studies), Canada [76] (one study), Russia [77] (one study), Taiwan [78] (one study), Belgium [79] (one study) and Chile [51] (one study). The sex of the animal model was mostly mixed in the studies, with 33 studies using both males and females, three studies used only males and four studies used only females. In the remainder (13 studies), the sex of the animals was not stated. 42 studies used the mouse *SOD1* ^*G93A*^ mouse model and four studies used the SOD1^G93A^ rat homologue. Other SOD1 mouse models included *SOD1* ^*G86R*^ *and loxSOD1* ^*G37R*^. Six studies used TDP‐43 knockout/mutant models, with mutations including *A315T*, *G348C* and *M337V*. The final mouse model that was used was the C57BL6/N model, which is a model for ageing. This mouse exhibits neuromuscular junction (NMJ) abnormalities and decreased motor function [80].

**TABLE 1:** Characteristics from the included studies.

Study	Animal info	Age	Delivery method	Dosages	Controls
SOD1 KO mice					
[35]	*SOD1* ^*G93A*^ mice	P2	ICV injection of AAV9 vector	1.4 × 10^14^/mL	Wild‐type mice (untreated) *SOD1* ^*G93A*^(untreated) *SOD1* ^*G93A*^ (AAV‐miR ctrl)
[36]	H11. Cas9 *SOD1* ^*G93A*^ mice	P0	Neonatal ICV, IT and IV (tail vein) injection of AAV	1 × 10^12^ vg/mouse	WT (*sgLacZ*) SOD1^G93A^ (*sgLacZ*)
[37]	*SOD1* ^*G93A*^ mice	P56–60	IT injection of AAV9 vector	8 × 10^10^ vg	Nontargeted control mice Uninjected mice
[38]	*SOD1* ^*G93A*^ mice	P0 and P50	Direct injection of AAV10 into spinal cord—P50 ICV and IV injection of AAV10—on P0	P50—9.5 × 10^10^ vg P0—7.8 × 10^11^ vg	SOD1^G93A^ with control antisense vector (AAV10‐U7‐CTR) Noninjected WT mice
[39]	*SOD1* ^*G93A*^ mice	P2	IM injections of AAV6/9 vectors	2.4 × 10^12^ vg	SOD1^G93A^ (scramble miR) SOD1^G93A^ (PBS‐treated) Wild type (PBS‐treated)
[40]	*SOD1* ^*G93A*^ mice	P0	ICV injection of AAV9 vector	4‐μL AAV‐RNAi in PBS	SOD1^G93A^ (vehicle‐treated)
[41]	*SOD1* ^*G93A*^ mice	P0–1	ICV injection with AAV9	1 × 10^11^ vg	Untreated *SOD1* ^*G93A*^ mice, WT mice and AAV9‐treated WT mice
[42]	*SOD1* ^*G93A*^ mice	P0	ICV injection of AAV9 vector	3 × 10^13^ vg/mL or 1 × 10^14^ vg/mL	LacZ sgRNA (negative control)
[43]	*SOD1* ^*G93A*^ mice	P0–1	IV (facial vein)injection with AAV9 vectors	4 × 10^11^ vg	Untreated and AAV9‐GFP injected mice
[44]	High‐copy *SOD1* ^*G93A*^ mice	P50–68	IV (tail vein) injection of rAAVh10	2 × 10^11^ vg	GFP vector and untreated *SOD1* ^*G93A*^ mice
[45]	*SOD1* ^*G93A*^ mice	P60	Intralingual (tongue) and intrapleural (near the lungs) injection of AAVrh10	Total dosage of 2 × 10^11^ vg per animal	Nontransgenic mice injected with PBS and untreated *SOD1* ^*G93A*^ mice
[46]	*SOD1* ^*G93A*^ mice	P30	IT injection of AAVrh10 vectors	3 × 10^12^ vg/mL	PBS‐injected transgenic and nontransgenic mice
[47]	*SOD1* ^*G93A*^ mice	P42	Intralingual injections of AAVrh10 vectors with treatment or saline	1 × 10^11^ vg	Saline injected and WT mice
[48]	*SOD1* ^*G93A*^ mice	P1 and P40	IT injection of scAAV9 vectors	Total dose of 5 × 10^10^ vg/g	Scrambled control vectors
SOD1 in combination					
[49]	*SOD1* ^*G93A*^ mice	P1old	ICV injection of AAV9 vectors into cerebrospinal fluid (CSF)	7.5 × 10^10^ vg	Untreated *SOD1* ^*G93A*^ mice
[50]	*SOD1* ^*G93A*^ mice + CDF1R‐cre+ (immune dysfunction) mice	P1	IV injections of AAV9 vectors to traverse the BBB	5 × 10^11^ vg	Untreated control mice
TDP‐43 KO mice					
[51]	*TARDBP* KO mice	P42	IV (tail vein) injection of AAV	1 × 10^12^ vg/mL	Control mice as heterozygous TDP‐43 KO had no effect
[52]	*TDP‐43* ^*G348C*^ and *TDP‐43* ^*A315T*^ mice	P240	Intracortical injections of scAAV2/9	9.2 × 10^9^ vg	scAAV2/9‐*GFP* vectors and PBS‐injected nontransgenic mice
[53]	*TDP‐43* ^*M337V*^ mice	Not stated	Stereotaxically injected with AAV8 vector into both sides of striatum—1 day following this, they were IV administered negative control or treatment	2 × 10^10^ vg (stereotaxic) and 1 × 10^11^ vg (IV)	Scrambled RNA and *GFP* and *RFP* vector controls
SOD1 KO mice + AAV encoding neurotrophic factors					
[54]	*SOD1* ^*G93A*^ mice	P42	IV and IM injection of AAV	2 × 10^12^ vg	AAV‐*Luc* vectors
[55]	*SOD1* ^*G93A*^ mice	P100	Intraspinal injections of AAV2 vectors	2.6 × 10^10^, 6.5 × 10^9^, 1.6 × 10^9^ vg/mouse	AAV2‐*GFP* vector and vehicle injected mice, uninjected mice also
[56]	*SOD1* ^*G93A*^ mice	P35	IV injection of AAV8 vectors	1.8 × 10^14^ vg/kg	AAV8 empty vectors and wild‐type mice
[57]	*SOD1* ^*G93A*^ rats	P175	IV (tail vein) injections of AAV9 vectors	100 μL containing 4 × 10^12^ DNase‐resistant particles	Empty AAV9 vector and saline injected
SOD1 KO mice + cells expressing neurotrophic factors					
[58]	*SOD1* ^*G93A*^ mice	P203	Injections of hUCBMC cotransduced with adenovirus 5 (Ad5) retro‐orbitally	2 × 10^6^ genetically engineered hUCBMC	Saline injected mice
[59]	*SOD1* ^*G93A*^ rats	P21–23	Injected with iNPCs into three sites in the left lumbar spinal cord	2 μL of iNPC‐GDNFs at 15,000 cells/μL	Untreated
[60]	*SOD1* ^*G93A*^ rats	~P80	Bilateral injections of neural progenitor cells expressing GDNF	Total of 400,000 cells per rat	NPC.WT and noninjected control transgenic rats
TDP43 KO mice + other growth factors					
[61]	*TDP‐43* ^*A315T*^ mice	P60	IT injection of rAAV9 vectors	1 × 10^9^ vg/mouse	Empty AAV9 vector, untreated transgenic and WT mice treated with both vector types
[62]	*SOD1* ^*G93A*^ mice	P60 and P90	Bilateral IM injections with AAV9 vectors	1 × 10^9^ vg/g	AAV9‐sgRNA‐*LacZ* and AAV9‐*GFP* vectors
[63]	*SOD1* ^*G93A*^ mice	P90	IT injection of scAAV9 vectors	1 × 10^12^ vg/mL	scAAV9‐*GFP* vectors and nontransgenic mice
[64]	*SOD1* ^*G93A*^ mice	P90	IV (tail vein) injection of scAAV9 vector	1.5 × 10^11^ vg	scAAV9‐*GFP* and nontransgenic mice
[65]	*SOD1* ^*G93A*^ mice	P60	IT injection of rAAV1 vectors	1 × 10^12^ vg/mL	AAV‐empty vectors and nontransgenic mice
[66]	*SOD1* ^*G93A*^ mice	P28	Single bilateral IM injection of LV	1 × 10^9^ TU/mL	LV‐*GFP*, nontransduced and untreated mice
Neuromuscular junction and synaptic support					
[67]	*SOD1* ^*G93A*^ mice	P90	IV (tail vein) injection of AAV9 vector	1.2 × 10^12^ vg	WT mice with and without vector injection
[68]	C57BL6/N mice exhibit NMJ abnormalities + decreased motor function	P730	IV (tail vein) injection of AAV9 vectors	4.8 × 10^13^ vg/kg	Empty AAV vectors
[69]	*SOD1* ^*G93A*^ mice	Not stated	IM injection of AAV1 into gastrocnemius muscles	1 × 10^11^ vg	Mock‐ or AAV1‐*GFP* vector and WT mice
[70]	*SOD1* ^*G93A*^ mice	P42	IV (tail vein) injection using AAV2/8 vectors	7.6 × 10^13^ vg	WT mice with and without empty AAV2/8 vectors
[71]	*SOD1* ^*G93A*^ mice	P49	IV (tail vein) injection using AAV8 vector IT injection using AAVrh10 vector	1 × 10^12^ vg for combined; 1 × 10^11^ vg for individual	Nontransgenic WT mice with and without mock *NRG‐III* and *NRG‐I* vectors
[72]	*SOD1* ^*G93A*^ mice	P60	IM injection in hind limbs and forelimbs using AAV‐DJ/8 vectors	8 × 10^10^ vg/mouse	Control WT mice with AAV‐*GFP*
[73]	*SOD1* ^*G93A*^ mice	P80	Bilateral IM injection into hindlimb quadriceps and thoracic muscles with AAV9 vectors	11 × 10^11^ vg	Injection of an AAV9::null vector and WT mice
[74]	*SOD1* ^*G93A*^ mice and rats	Not stated	Lumbar subpial vector injection of scAAV9 vectors	2 × 10^13^ vg/mL	WT mice, untreated and null‐/saline‐treated transgenic mice
[75]	*SOD1* ^*G93A*^ mice	P30	Intraspinal injection of AAV8 vectors. IV (tail vein)injections of AAV vectors also occurred	4 × 10^12^ vg/mL	WT mice injected withAAV‐*GFP* and saline injections
Autoinflammation/immune modulation					
[76]	*SOD1* ^*G93A*^ mice	P0–6 h	Intraspinal injections of rAAV2/1, 2/5, 2/8 and 2/9 vectors	1 × 10^3^–1 × 10^7^ copies/mL	Uninjected mice and AAV2/1‐*GFP* vectors
[77]	*SOD1* ^*G93A*^ mice	P30	Injection within the cisterna magna of lentivirus	10^7^ Plaque forming units	Lentivirus with *GFP*
[78]	h*SOD1* ^*G93A*^ and lox*SOD1* ^*G37R*^ mice	P1	Intraspinal injection of scAAV2/9 vectors	5.35 × 10^8^ vg	AAV2/9‐*GFP* and uninjected mice
Glutamate regulation and excitotoxicity control					
[79]	*SOD1* ^*G93A*^ mice	P110	Intraspinal injection of AAV8 into the spinal cord	1 × 10^13^ vg/mL	AAV vectors containing *eGFP* were
[80]	*SOD1* ^*G93A*^ mice	P10	ICV injections of AAV9	1 × 10^11^ vg per mouse	Uninjected *SOD* ^*G93A*^, AAV9‐*GFP*, FLAG/Myc and empty. *SOD1*.WT mice
[81]	*SOD1* ^*G93A*^ mice	P90	IT injection of AAV9 vectors	1 × 10^12^ vg/kg	AAV9‐*GFP* vector and nontransgenic mice
[82]	*SOD1* ^*G93A*^ mice	P65	Intracisternal and IM injections of lentivirus	5 × 10^8^ vg	Saline and LV‐*GFP* injections
[83]	*SOD1* ^*G93A*^ mice	P56	Injected from tail vein with bone marrow cells after lentiviral infection	100 viral particles/cell	Empty LV vectors, with GFP And uninjected *SOD1* ^*G63A*^ mice
Cellular stress response					
[84]	*SOD1* ^*G86R*^ mice *SOD1* ^*G93A*^ mice *TDP43* ^*A315T*^ mice	P0–2	ICV injection of AAV2 vectors	2 × 10^10^ vg	Nontransgenic mice and AAV‐MOCK vectors
[85]	TDP‐43 lesion rat model—AAV9 was used to transduce rats with lesions	Unsure	IV (tail vein) injections of AAV9 vectors	1 × 10^12^ vg	AAV9‐*GFP* and uninjected mice
[86]	*SOD1* ^*G93A*^ mice	P30	IT injection using AAV9 vector	3.61 × 10^13^ vg/mL	AAV‐*GFP*
[87]	TDP‐43 transgenic mice	P1	Bilateral cerebral ventricle injection with AAV9 vectors	2 × 10^10^ vg/pup	Nontargeting and wild‐type controls

Abbreviations: AAV = adeno‐associated vector; Ad5 = adenovirus 5; GDNF = glial‐derived neurotrophic factor; hUCBMC = human umbilical cord blood mononuclear cells; ICV = intracerebroventricular; IM = intramuscular; iNPC = inducible neural progenitor cells; IT = intrathecal; IV = intravenous; KO = knock out; LV = lentivirus; P = postnatal; WT = wild type.

The included studies have been grouped by the genetic background of the animal models used for ease of presentation; however, a stratified meta‐analysis based on genetic background has not been performed. For example, 14 studies reported genetic therapies in SOD1 mice [35, 36, 37, 38, 39, 40, 41, 42, 54, 55, 67, 69, 70, 81]. Other groups included *SOD1* in combination with either Galectin‐1 or NF‐κB [43, 44] (two studies), as well as *TDP‐43* [45, 76, 82] (three studies). However, the majority of studies were split into target genetic therapy group: neurotrophic factors such as BDNF, Neurturin and GDNF [46, 47, 61, 77, 83, 84, 85] (seven studies); other growth factors such as VEGF, HGF and IGF‐1 [48, 56, 57, 58, 65, 71] (six studies) or into the biological pathway that the gene is a part of. In this grouping, studies reviewed centred on NMJ and synaptic support focusing on Dok7, Nrg1, NRIP, SYT13, SynCav1 and Esyt1 [49, 62, 63, 64, 66, 72, 78, 80, 86] (nine studies) as well as autoinflammation/immune modulation targeting EGFP, IL‐4 and MIF [68, 73, 74] (three studies). Additionally, five studies looked at targets in glutamate regulation and excitotoxicity control (GLT1, MCT1, DAO and EAAT2) [50, 59, 75, 79, 87], and four studies targeted the cellular stress response (XBP1, OPTN, Ataxin‐2 and UPF‐1) [51, 52, 53, 60]. Additional information regarding the outcomes from the studies is presented in Tables S1 and S2.

### 3.3 Risk of Bias Assessment

**FIGURE 2:**
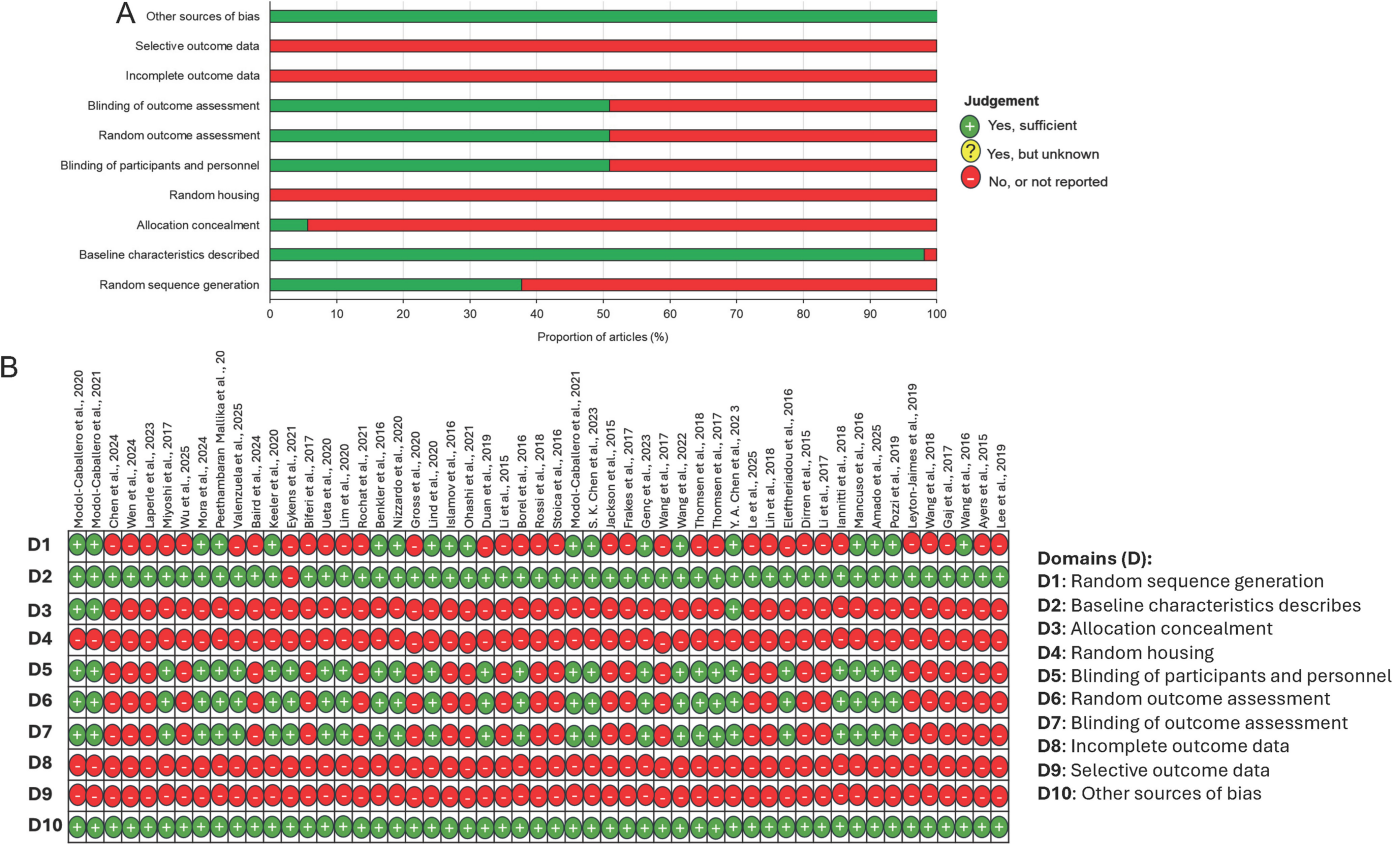
Risk of bias assessment. (A) Summary chart for risk of bias in all studies. (B) Risk of bias in individual studies for each domain.

Most studies had an overall high risk of bias (Figure 2), with significant proportion of studies (Figure 2A) presenting with high risk present in the most important individual domains (Figure 2B). For example, 18 out of 53 (34%) studies stated that they performed their genetic therapy injections blinded, whereas only 27 (51%) studies performed their experiment in a blinded fashion in order to remove bias in the analysis. Only 3 out of 53 (~6%) studies stated that they concealed the allocation of the therapy injected from the researcher performing the experiments. However, the majority of studies identified baseline characteristics in their studies and stated they were similar between groups in their study.

### 3.4 Study Outcomes

Our primary outcome for this systematic review was neuroprotection, with secondary outcomes for motor neuron cell survival and improvement of motor function in animals treated with specific genetic therapies. Numerical data used to conduct the meta‐analysis are presented in Table S2.

#### 3.4.1 Neuroprotection

**FIGURE 3:**
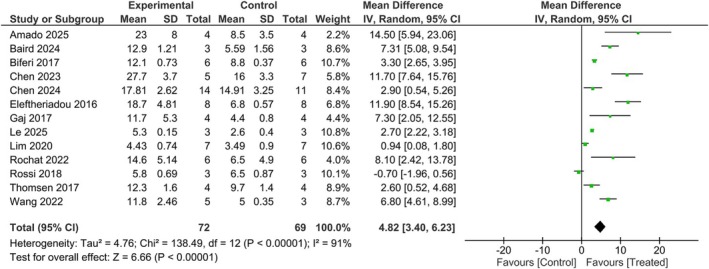
Forest plot to depict results from meta‐analysis of appropriate studies measuring neuroprotection with ChAT immunostaining. Studies presenting motor neuron survival counts were pooled together and means ± SD were used to calculate the mean difference and 95% confidence intervals as well as *p*‐value (test for overall effect). Black diamond in the Forest plot represents the overall mean favouring the treated group. Studies: Amado et al. [53]; Baird et al. [43]; Biferi et al. [69]; Chen et al. [35]; Chen et al. [78]; Eleftheriadou et al. [65]; Gaj et al. [39]; Le et al. [46]; Lim et al. [36]; Rochat et al. [67]; Rossi et al. [73]; Thomsen et al. [61]; Wang et al. [49].

For neuroprotection, most of the studies used Choline Acetyltransferase (ChAT) [35, 36, 39, 43, 46, 49, 53, 61, 65, 67, 69, 73, 78] or NeuN [57, 58, 59, 60] staining. A meta‐analysis of the pooled data from studies using ChAT showed a significant increase in the number of surviving neurons, with a mean difference of 4.06 ChAT^+^ cells per section (95% CI [2.83, 5.30]), favouring the genetic therapy treatment group (*p* < 0.00001). However, heterogeneity of the studies was high (*I* ^2^ = 92%, *χ* ^2^ = 186.16). A meta‐analysis of neuroprotection assessed using NeuN staining showed an overall mean difference of 2.74 cell per section (95% CI 2.74, 3.15), *p* < 0.00001, favouring the genetic therapy, with very small heterogeneity (*I* ^2^ = 0% and *χ* ^2^ = 0.41) (Figure 3).

#### 3.4.2 Animal Survival

**FIGURE 4:**
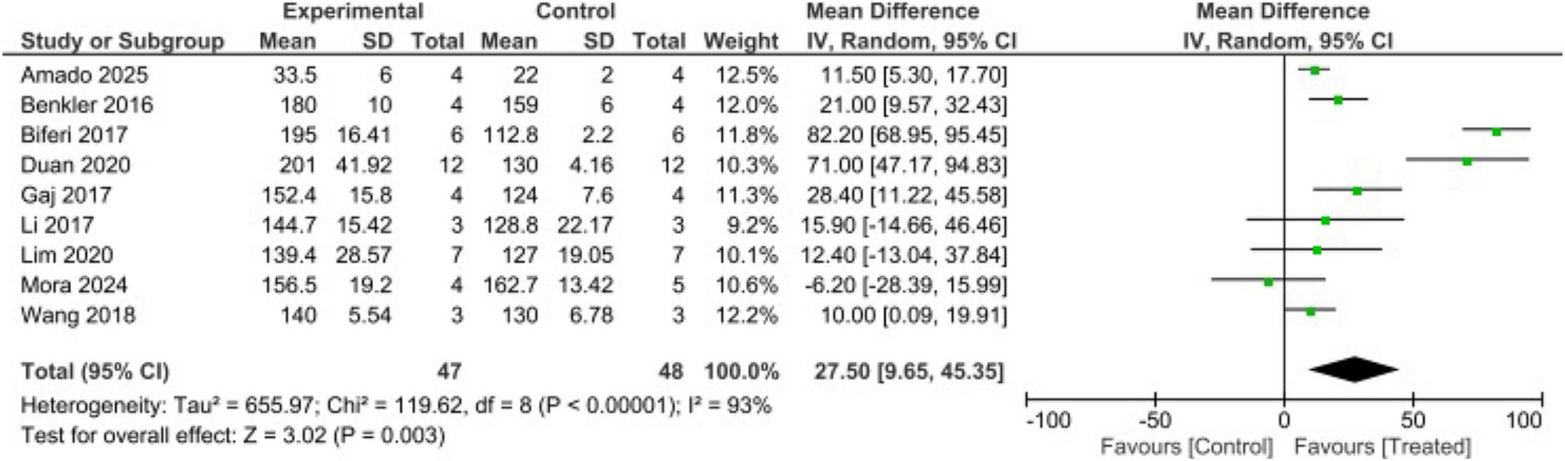
Forest plot to depict results from meta‐analysis of appropriate studies measuring overall animal survival after genetic therapy treatment. Only studies that presented mean survival ± SD were used to calculate mean difference and 95% confidence intervals as well as *p*‐value (test for overall effect). Black diamond in the Forest plot represents the overall mean favouring the treated group. Studies: Amado et al. [53]; Benkler et al. [75]; Biferi et al. [69]; Duan et al. [54]; Gaj et al. [39]; Li et al. [50]; Lim et al. [36]; Mora et al. [66]; Wang et al. [58].

ALS models treated with the control genetic therapy overall showed a significantly lower mean survival rate than those that were treated with the genetic therapy [39, 50, 53, 54, 58, 66, 69, 75]. A meta‐analysis of the pooled data for the studies that reported survival rates showed a significant increase in animal survival, with a mean difference of 27.5 days (95% CI [9.65, 45.35]) favouring the treatment group (*p* < 0.003) (Figure 4). However, an extremely high heterogeneity was also displayed in these studies (*I* ^2^ = 93%, *χ* ^2^ = 655.97). Additionally, there was a varied amount of significance in the individual study results, with 19 studies reporting statistically significant increase in survival and four studies reporting no significant differences after genetic therapy.

#### 3.4.3 Motor Function

Another outcome measured was motor function in animals quantified by measuring their ability to remain on a rotating rotarod [42, 53, 69, 70, 75, 78, 82].

**FIGURE 5:**
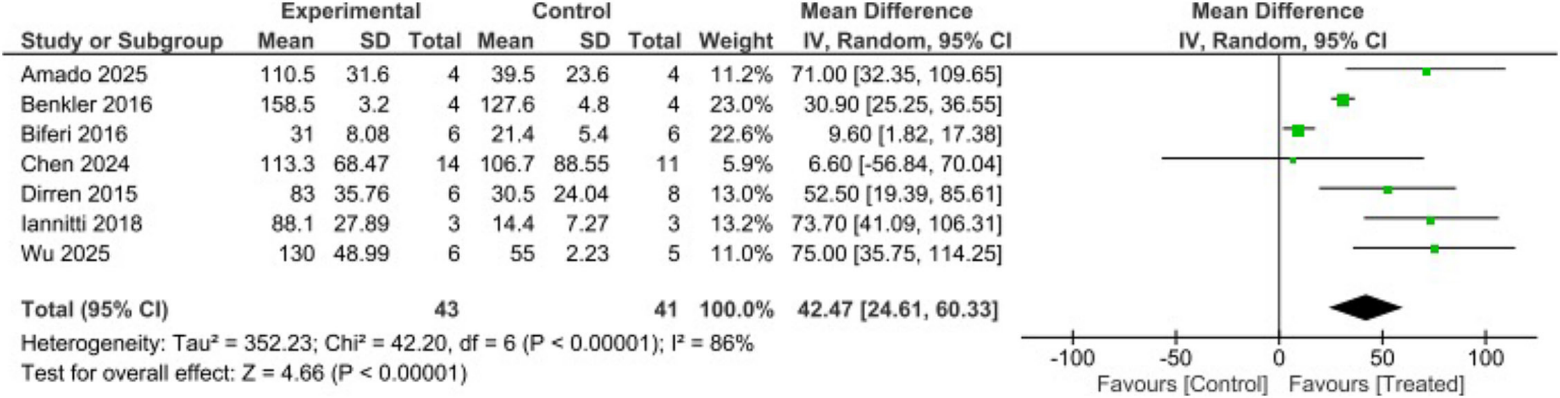
Forest plot to depict results from meta‐analysis of appropriate studies measuring rotarod performance. Studies presenting latency to fall were pooled together and means ± SD were used to calculate the mean difference and 95% confidence intervals as well as *p*‐value (test for overall effect). Black diamond in the Forest plot represents the overall mean favouring the treated group. Studies: Amado et al. [53]; Benkler et al. [75]; Biferi et al. [69]; Chen et al. [78]; Dirren et al. [70]; Iannitti et al. [42]; Wu et al. [82].

Our meta‐analysis showed a significantly increased motor performance time, with a mean difference of 42.47 s (95% CI [24.61, 60.33]), favouring the genetic therapy treatment (*p* < 0.00001). However, as with the motor neuron count and animal survival, the heterogeneity of this outcome was also large (*I* ^2^ = 86%, *χ* ^2^ = 352.23) showing a variation in the results of the studies (Figure 5).

## 4 Discussion

The objective of this study was to evaluate the efficacy of a variety of genetic therapies targeting ALS over the past 10 years. We identified 53 studies that met our inclusion and exclusion criteria. Over these studies, the experiments performed differed greatly, and from these studies, we chose the number of motor neurons (neuroprotection), the mean survival of the animal and motor performance on the rotarod as our main outcomes, because these were the outcomes that were commonly reported in many studies. The meta‐analyses for the three outcomes assessed all showed significant benefits after genetic therapy, demonstrating neuroprotection, animal survival and motor performance. However, given the overall risk of bias in the studies was judged to be high, caution must be exercised when drawing definitive conclusions about the benefits of genetic therapy in preclinical models of ALS.

ALS is a polygenic disorder, with as many as 40 genes now associated with the disease, including multiple mutations identified in several genes. This makes identifying a single genetic therapy that works for most patients much harder. Despite the many genes and mutations now associated with ALS, a large proportion of the studies reviewed here tested their therapies in similar transgenic animal models, predominantly the SOD1^G93A^ model. This means that these studies are limited to testing the outcomes in one subtype of ALS. However, the prevalence of SOD1‐causing ALS is only around 2.5% [88], and so the results from these studies are unlikely to have universal benefits. Only seven of the studies included in this systematic review used models that were not SOD1^G93A^ ALS, for example, TDP‐43. Six studies [45, 48, 52, 53, 76, 82] utilise TDP‐43 rodent models instead, showing promising results using these models. Interestingly, the accumulation of TDP‐43 is the most significant pathological outcome in around 95% of ALS cases [89]. This implies that the *TARDBP* gene, encoding for the TDP‐43 protein may be a more universal target for ALS therapies. Other models could also be used, for example, C9orf72 hexanucleotide expansion models, and these will help to create a broader picture of how genetic therapy can affect ALS patients with other variants, especially as the C9orf72 expansion is the most commonly seen genetic aberration in ALS.

The heterogeneous genetic nature of ALS that will likely limit a genetic therapy to only a specific group of patients explains why some groups attempted to develop therapies that would target common pathways rather than ALS‐associated genetic lesions per se. For example, neurotrophic and growth factors support motor neuron survival and therefore manipulating them to increase neuroprotection could be a beneficial avenue to explore for multiple ALS patients [90]. These factors were tested in 13 trials (Table 1). Inflammation is a component of all neurodegenerative diseases, and so reducing neuroinflammation is another potential avenue through which a genetic therapy could be used to treat the disease [91]. This was tested in three studies. Glutamate regulation is severely impaired in ALS, and glutamate itself is involved in the degeneration of motor neurons [92]. The first ever approved ALS drug, Riluzole, plays a role in reducing glutamate‐based neurotoxicity, so there is promise in this pathway for potential drug therapies. Glutamate regulation was tested in five studies. Finally, stress within the cell has been found to trigger the death of motor neurons in ALS. Therefore, targeting stress responses and regulating them could be a beneficial, more universal mechanism for treatment [93]. This was tested for in four studies. With the exception of two studies targeting glutamate regulation [50, 79], in each of the trials targeting common pathways, benefits were seen, and this approach may prove to be of more universal benefit to patients than therapies targeting specific ALS‐associated genes [59, 75, 87].

From the results of this review of preclinical studies, it can be concluded that genetic therapy has the potential to benefit ALS patients. Motor neuron count, survival and muscle function all displayed significant improvements favouring intervention when compared to controls, particularly in studies using SOD1 as the target. However, these results should also be viewed with caution. Firstly, the risk of bias across these studies was judged as high, mainly due to a lack of clarity about how some studies were conducted. This could indicate flaws in those studies: Inadequate blinding of sequence generation—the random allocation of the animals to their experimental groups—and outcome assessment are important domains containing high risk of bias in many studies. Additionally, the heterogeneity of each meta‐analysis showed that results varied greatly. This is due partly to the fact that studies used a large variety of gene targets and methods of delivery. Another likely reason for the heterogeneity is due to a failure to standardise methodology for phenotyping animals and for quantification of outcomes such as motor neuron survival.

Notably, the results from these studies show that the method of delivery—intracerebroventricular, intrathecal or intravenous—did not have much impact on the success of the therapy. The therapies that proved unsuccessful [50, 79] were administered by intraspinal and intracerebroventricular injections, respectively. However, other studies in this review used the same delivery methods with positive outcomes. Similarly, the vector used did not seem to influence the outcomes significantly: The two studies that showed no beneficial effect of the therapy used AAV [50, 79]. However, this delivery method was also used in many of the studies that did report a beneficial effect. Four studies [65, 73, 75, 87] used lentiviruses as the method of delivery, with varying levels of success. Other methods of delivery included transfected neural progenitor cells (NPCs), used in two studies [84, 85], as well as human umbilical cord blood‐derived mast cells (hUCBMCs), used in one study [77].

Another limitation of this study comes with the difficulties of transferring animal model experiments to human samples or clinical trials [94]. In order to improve success before any progress into clinical trial, therapies should be tested over multiple preclinical models, like human tissues and cell lines. No singular model can fully emulate a human disease model, but the more models tested on before being advanced, the more translatable the results are likely to be.

A bias that is inherent to preclinical studies is a limitation for this review. It is more likely that a study with a positive outcome will be published than one in which the tested therapy showed no, or a negative, benefit and some unsuccessful studies are likely, therefore, to remain unpublished. This contrasts with clinical trial results which are usually published regardless of the outcome. This bias towards positive publications leaves valuable knowledge of gene targets, vectors or methods residing only within a particular group, to the detriment of the field. More acceptance of well‐performed but negative studies would potentially drive progress towards a successful therapy [95]. Nevertheless, two studies included in this systematic review [50, 79] did publish results reporting that their genetic therapy did not provide any therapeutic benefit.

## 5 Conclusions

This systematic review highlights the potential for genetic therapies in ALS, as well as the necessity for more research into the disease, particularly to target ALS‐associated genes beyond SOD1. Interventions in preclinical rodent models have proved positive and suggest a promise for the future development of ALS treatments. However, this review has limitations and results should be taken with caution. The heterogeneity of the meta‐analysis, even within the same gene targets, reveals high variability between the different therapies and methods that is likely to be enhanced by the complexity of ALS. Efforts to standardise delivery and phenotyping methods would reduce this heterogeneity. In addition, studies should expand genetic therapy testing into other models of ALS to provide greater supporting evidence for the efficacy of the therapy and to derisk clinical trials that go on to test the therapy in ALS patients.

## Author Contributions

H.E.W. made substantial contributions to concept and design, acquisition of data, analysis and interpretation of data, as well as writing the manuscript. Z.A. made substantial contributions to conception, meta‐analysis and interpretation of data, as well as revisions of the manuscript. R.I.T. made substantial contributions to conception and revisions of the manuscript. All the authors have read and approved the final version of the manuscript.

## Funding

H.E.W. is funded by studentship through the LifeArc Centre for Acceleration of Rare Disease Trials (ARDT). The ARDT is a collaboration between Newcastle University, Queen's University Belfast, and the University of Birmingham. The centre is funded/supported by LifeArc (under grant no. 10747). LifeArc is a charity (registered in England and Wales under no. 1015243 and in Scotland under no. SC037861). The opinions and interpretations presented herein are those of the authors and not LifeArc's.

## Ethics Statement

The authors have nothing to report.

## Conflicts of Interest

The authors declare no conflicts of interest.

## Supporting information


**Table S1:** Additional information on characteristics of the included studies.
**Table S2:** Outcomes from the included studies.
**Table S3:** Numerical data used for meta‐analysis.
**Figure S1:** Forest plots to demonstrate results of meta‐analysis stratified by delivery method. Forest plots show changes in ChAT^+^ motor neurons when delivered by (A) ICV and (B) IM.
**Figure S2:** Forest plots to demonstrate results of meta‐analysis stratified by delivery method. Forest plots show changes in NeuN^+^ motor neurons when delivered by IT.

## Data Availability

The data and materials used to support the findings of this study are available from the corresponding author.
