# Supplementary material for: Exploring Genetic Therapies Targeting Amyotrophic Lateral Sclerosis in Animal Models: A Systematic Review and Meta‐Analysis
Source: J Gene Med. 2026 Aug 18;28(8):e70106. doi: 10.1002/jgm.70106 (PMC13482302; doi:10.1002/jgm.70106)
Supplement: Supplementary file 1 [file JGM-28-e70106-s001.docx]

**SUPPLEMENTARY MATERIALS**

**Exploring gene therapies targeting Amyotrophic Lateral Sclerosis in animal models: a systematic review and meta-analysis**

Hannah E. Wedgwood^1,2^, Richard I. Tuxworth^2,4^, Zubair Ahmed^1,3,4,^*

^1^Department of Inflammation and Ageing, School of Infection, Inflammation and Immunology, University of Birmingham, Birmingham, United Kingdom

^2^Department of Cancer and Genomic Sciences, School of Medical Sciences, College of Medicine and Health, University of Birmingham, Birmingham, United Kingdom

^3^University Hospitals Birmingham NHS Foundation Trust, Mindelsohn Way, Birmingham, United Kingdom

^4^Centre for Neurogenetics, University of Birmingham, Birmingham, United Kingdom

***Correspondence to:** Professor Zubair Ahmed, [z.ahmed.1@bham.ac.uk](mailto:z.ahmed.1@bham.ac.uk)

**Supplementary Table 1.** Additional information on characteristics of the included studies.

| Study | Study origin | Sample size | Sex |
| --- | --- | --- | --- |
| SOD1 KO mice | | | |
| ^36^ | Switzerland/ France | n = 4-12 / group | Not stated |
| ^37^ | USA | n = 10 / group | Not stated |
| ^38^ | USA | n = 3-15 / group | Males/ Females |
| ^39^ | France | n = 2-25 / group | Sex-balanced |
| ^40^ | Switzerland | n = 3-15 / group | Not stated |
| ^41^ | USA | n = 3-13 / group | Not stated |
| ^42^ | USA | n = 4-22 / group | Not stated |
| ^43^ | China | n = 4-11 / group | Males/ Females |
| ^44^ | USA | n = 3-7 / group | Males/ Females |
| ^45^ | USA | n = 7-22 / group | Males/ Females |
| ^46^ | USA | n = 2-8 / group | Mostly females – 1 male for each condition |
| ^47^ | China | n = 3-4 / group | Males/ Females |
| ^48^ | USA | n = 6-11 / group | Males/ Females |
| ^49^ | UK/USA | n = 3-15 / group | Not stated |
| ^50^ | USA | n = 3-10 / group | Males/ Females |
| SOD1 in combination | | | |
| ^51^ | USA | n = 8-33 / group | Not stated |
| TDP-43 KO mice | | | |
| ^52^ | USA | n = 2-20 / group | Males/ Females |
| ^53^ | Canada | n = 3-7 / group | Males/ Females |
| ^54^ | China | n = 4-8 / group | Not stated |
| SOD1 KO mice plus neurotrophic factors | | | |
| ^55^ | USA | n = 3-4 / group | Males/ Females |
| ^56^ | USA | n = 3-12 / group | Not stated |
| ^57^ | Spain | n = 5-13 / group | Males/ Females |
| ^58^ | USA | n = 4-10 / group | Males/ Females |
| ^59^ | Russia | n = 3-11 / group | Males/ Females |
| ^60^ | USA | n = 4-10 / group | Male |
| ^61^ | USA | n = 2-16 / group | Males/ Females |
| TDP43 KO mice + other growth factors | | | |
| ^62^ | USA / South Korea | n = 3-7 / group | Females |
| ^63^ | China | n = 3-15 / group | Males/ Females |
| ^64^ | China | n = 5-15 / group | Males/ Females |
| ^65^ | China | n = 3-15 / group | Females |
| ^66^ | South Korea | Not stated | Not stated |
| ^67^ | UK | n = 2-24 / group | Males/ Females |
| Neuromuscular junction and synaptic support | | | |
| ^68^ | Japan | n = 3-24 / group | Males |
| ^35^ | Japan | n = 4-21 / group | Male |
| ^69^ | Spain | n = 3-20 / group | Males/ Females |
| ^70^ | Spain | n = 3-14 / group | Males/ Females |
| ^71^ | Spain | n = 3-6 / group | Females |
| ^72^ | Taiwan | n = 3-14 / group | Males/ Females |
| ^73^ | Italy | n = 3-15 / group | Males/ Females |
| ^74^ | USA/Japan | n = 3-19 / group | Males/ Females |
| ^75^ | UK | n = 2-12 / group | Males/ Females |
| Auto-inflammation/immune modulation | | | |
| ^76^ | USA | n = 3-16 / group | Males/ Females |
| ^77^ | Italy | n = 3-12 / group | Not stated - Males were used for BM transplants |
| ^78^ | Israel | n = 3-20 / group | Not stated |
| Glutamate regulation and excitotoxicity control | | | |
| ^79^ | USA | n = 3-5 / group | Males/ Females |
| ^80^ | Belgium | n = 3-26 / group | Males/ Females |
| ^81^ | China | n = 3-30 / group | Males/ Females |
| ^82^ | Israel | Not stated | Males/ Females |
| ^83^ | Japan | n = 5-15 / group | Not stated |
| ^84^ | Chile/USA | n = 3-22 / group | Males/ Females |
| ^85^ | USA | n = 3-7 / group | Males/ Females |
| ^86^ | China | n = 3-10 / group | Females |
| ^87^ | USA | n = 3-40 / group | Males/ Females |

**Supplementary Table 2.** Outcomes from the included studies

| Study ID | Gene therapy | Behavioural results | Neuroprotection | Significance | Side effects |
| --- | --- | --- | --- | --- | --- |
| SOD1 KO mice | | | | | |
| (Rochat *et al.*, 2022) ^36^ | microRNA delivering RNAi against SOD1 | Muscle strength and rotarod tests showed a recovery of motor function following treatment | MN numbers were increased from control flies by late/end-stage of the disease | Mildly significant; Significance was increased over time following the aging of the mice and the development of the disease, for all tests (from ns to ***) | No side effects were observed/ tested |
| (Y. A. Chen *et al.*, 2023) ^37^ | AAV-PHP.B variants to deliver CRISPR-Cas9 guide RNAs, to disrupt the human SOD1 | Survival, body weight, rotarod, clasping tests, total distance and latency to fall tests show improvements following treatments | Injection of AAV-sgSOD1 prevents NMJ loss and neurodegeneration | Significant differences between treated and untreated SOD1*G93A mice. This study shows a lack of statistical differences between WT mice and treated mice, showing a recovery of muscle and neuron function. | No off-target effects were seen in human HeLa cells, but were not mentioned in the mice studies. |
| (Lim *et al.*, 2020) ^38^ | Delivery of cytidine base editors (CBEs) | Survival, disease length, rotarod and body weight were all measured and had a positive effect following treatment | MN numbers were increased as was the survival of NMJs. | Significance varied between tests; Behavioural tests show changes in significance over time (ns up to ***). MN numbers and NMJs are recovered slightly, with a small significance (*) | No side effects were observed/ tested |
| (Biferi *et al.*, 2017) ^39^ | Introduction of U7-hSOD1 throughout the mice | Neuromuscular function was preserved, tested by body weight, strength coordination and spontaneous activity assessments | Motor neuron and microglia numbers increased following therapy | Significant; Behavioural tests show significant results (** to ***) across the different assays. Motor neuron numbers increased significantly compared to the control (****), as did the microglia cell numbers (****) | No adverse effects of this treatment have been found |
| (Dirren *et al.*, 2015) ^40^ | AAV6/9 vectors encoding microRNA against human SOD1 | Compound muscular action potential (CMAP) was greatly improved, as was swimming latency and survival | Motor neurons were measured per section, and were increased compared to controls, as was the % of occupied NMJs. | Significant; For CMAP and swimming latency, the treated mice showed similar results to the WT mice, indicating a large recovery, and a large difference compared to control (***). Motor neuron numbers were lower than WT mice but significantly different to controls (***). | Some animals displayed systemic abnormalities: signs of ataxia, increased susceptibility to eye and skin infections and gastrointestinal complications |
| (S. K. Chen *et al.*, 2023) ^41^ | AAV9 vector with miRNA targeting SOD1 – different doses tried | ALS-like phenotypes are reduced following treatment, including early deaths and pNF-H levels | No MN number tests were performed, so neuroprotectivity cannot be deduced | Mild significance; Survival was improved following treatment of miR30a-SOD1 (*). pNF-H concentration was significantly reduced (***) | Adverse effects were identified with some, but no all, variations of treatments. miR30a treatment had much less adverse effects. |
| (Stoica *et al.*, 2016) ^42^ | AAV9 vector with miRNA against hSOD1 | Treatment improves neuromuscular function and increases lifespan | Survival of spinal cord and corticospinal motor neurons | Significant; Survival was increased by 69 days following treatment and electrophysical tests show a preservation of MN function following treatment (*). MN numbers are recovered following treatment (* to **) | Off-target effects were assessed for and no significant changes were observed |
| (Duan *et al.*, 2020) ^43^ | CRISPR/CAS9 delivered by AAV against SOD1 | Stride length, latency to fall and survival/weight tests showed an improvement following treatment | The number of motor neurons is greatly increased following treatment in affected mice | Significant; Mice have a much larger chance of survival (71 days), a much larger stride length (**) and a significant increase in weight. Motor neurons increased by about 3x following treatment (***) | No off-target effects in the genome occurred from the treatment |
| (Gaj *et al.*, 2017) ^44^ | CRISPR-Cas9 harnessed to disrupt mutant SOD1 expression | Disease onset, survival, body weight and rotarod tests were used to demonstrate functional recovery following treatment | Lumbar and thoracic spinal cord motor neurons were counted per section, and results showed an increase in numbers following treatment | Significant; Behavioural tests all show increase in function. Disease onset, survival, weight and rotarod tests show significant recovery (***). Lumbar and thoracic motor neuron numbers are increased compared to untreated (* to **). | No side effects were mentioned or observed. |
| (Borel *et al.*, 2016) ^45^ | Silencing of SOD1 | Survival, disease onset/duration showed an increase. Similarly, grip strength, latency to fall and respiratory tests were improved | Motor neuron numbers were not assessed | Significant; Grip strength had a very significant change following treatment (****), latency to fall and respiratory tests also showed significant differences (* to **). Survival was increased by 27 days, and the disease onset was increased by 31 days (****) | No toxicity was observed during these tests following treatment |
| (Keeler *et al.*, 2020) ^46^ | miRNA targeting (superoxide dismutase 1) SOD1 protein | Increased survival/weight, improved function of strength and breathing | Motor neuron death is observed in both treated and control mice | Mildly significant; body weight assays showed good significance, however less significance was shown in the other behavioural tests. Breathing was improved (P<0.05). Motor neuron survival was assessed but no improvement was observed | No side effects mentioned |
| (Li *et al.*, 2017) ^47^ | Recombinant AAV delivering SOD1-RNAi at fast/slow injection speeds | Disease onset, survival, weight and footprint length show an improvement with both treatments, but more with slow injections | Number of motor neurons were measured with slow injected treatment and showed an improvement but not a full recovery. | Significant; No statistical analysis was performed for disease onset and survival, but for footprint length (**) and weight (*) a significant improvement was observed. For the number of neurons, the slow treatment was tested singularly and showed a statistical improvement from control SOD1 mice (**) but still a decrease from WT (**). | No side effects were mentioned or observed. |
| (Lind *et al.*, 2020) ^48^ | miRNA targeting SOD1 | Respiratory function was improved following treatment, as well as survival of the mice. Swallowing function was also tested but not improved | Motor neuron survival was not assessed | Low significance; Mean inspiratory flow was improved following treatment compared to controls in normal ventilation. Survival was increased significantly for female mice (p<0.05). Swallowing function was not significantly improved. | Only the female mice showed any effect in the survival assays; Fewer negative impacts using this method relative to shRNAs |
| (Iannitti *et al.*, 2018) ^49^ | Using AAV9 to deliver RNAi against hSOD1 | Body weight, latency to fall, neuroscoring, survival and disease onset all improved following treatment, shown at different post-natal days. Stride length and swing speed was also improved | Motor neuron survival was increased following treatment | Significant; All behaviour tests showed significant improved in motor and cognitive function at post-natal day 1 (** to ****) and post-natal day 40 (** to ****). Motor neuron survival was increased, with slight significant (*) difference between the treatment and control vectors | No significant off-target effects were shown to be linked to the methods used. |
| SOD1 in combination | | | | | |
| (Baird *et al.*, 2024) ^50^ | Combination therapy with galectin-1 and SOD1 downregulation | Rotarod and grip strength tests showed improvement of motor functions. Survival assays also showed positive results | Motor neuron survival was increased, as well as the total area of motor neurons | Significant; Survival assays showed P<0.0001. Statistical analysis was not performed on the other motor tests. Motor neuron survival was very significant (****) | Treated mice experienced kyphosis (curvature of the spine), lethargy (fatigue) and dramatic weight loss |
| (Frakes *et al.*, 2017) ^51^ | Suppression of microglial NF-κB and SOD1 expression in astrocytes and MNs | Body mass, disease onset/progression, rotarod and grip strength tests show a recovery of motor function following treatment. | No motor neuron number tests were performed. | Significant; A combinational approach (targeting astrocytes, MNs and microglia) was found to be most effective in increasing lifespan (****). Disease onset and progression was also found to be best improved via a combination treatment (*** to ****) | No toxic effects were reviewed in this study |
| TDP-43 KO mice | | | | | |
| (Peethambaran Mallika *et al.*, 2025) ^52^ | Expression of splicing repressor CTR | Grip strength tests showed improvements compared to controls, similar to the body weight and survival analysis | Motor neuron loss, axon degeneration and skeletal muscle atrophy was attenuated | Significant; Grip strength and body weight analysis showed significant results (P<0.0001). Similarly, the neuron survival showed significance (****) | No adverse effects found after long-term exposure |
| (Pozzi *et al.*, 2019) ^53^ | Single-chain antibodies against RNA recognition motif 1 (RRM1) of TDP-43 | Cognitive tests (% of time spent exploring objects) with familiar and novel objects show and improvement following treatment compared to controls | The complete % of total NMJs was increased in comparison to a control vector | Significant; Cognitive tests were performed at 2 months and 4 months, showing significant difference between familiar and novel objects at both (***), but 4 months showed more similar results to WT mice. | No side effects mentioned, only beneficial effects have been found from this drug. |
| (Wu *et al.*, 2025) ^54^ | siRNAs targeting TAR DNA-binding protein-43 (TDP-43) | Motor function was improved following IVSA-siR-TDP43 – tested via balance beam, rotarod and grip strength tests | Neuronal loss was reduced following IVSA-siR-TDP43 therapy, as well as a reduction in astrocyte and microglia in diseased mice | Significant; Behavioural tests show good significance compared to IVSA-scramble (* to ****). Neuronal protection was significant also (** to ****) | There were no toxic side effects but needs to be monitored; TDP-43 loss has shown potential toxic |
| SOD KO mice + AAV encoding neurotrophic Factors | | | | | |
| (Le *et al.*, 2025) ^55^ | AAV vectors delivering BDNF or GAS6 to muscles | Disease onset was improved following treatment individually. Progression of disease was monitored using neurological tests and rotarod tests. | The number of motor neurons increased following treatment, and improved as time increased post-treatment | Significant ; Authors show a range of significances in the results in the behavioural tests following treatment compared to control mice (ns to ****). The significance of motor neuron differences increased over time: 75 days (ns), 115 days (*), end-stage (** and ****) | Previous studies of both delivery methods showed no adverse effects |
| (Gross *et al.*, 2020) ^56^ | Neurotrophic factor (Neurturin) –> dose-dependent tests | Forelimb grip strengths were improved, however weight and survival were not affected as much | Motor neuron numbers were increased depending on dose of treatment | Mild significance; forelimb grip strength tests showed some significant improvement for all doses (*), but the other behavioural tests showed no significance. MN numbers increased with the dosage of Neurturin (ns to ****) | Off-target transduction of cells in areas outside the neuraxis could be a potential side effect |
| (Mòdol-Caballero, García-Lareu, *et al.*, 2021) ^57^ | AAV vectors coding for GDNF under the control of the desmin promoter | Body weight showed some improvement following treatment. Electrophysical tests show more of a positive effect. | Increased GDNF signalling preserves NMJ and activates pathways in muscles | Mild significance; body weight shows a small difference between treatment and control (up to *) and electrophysical tests show more differences (*). NMJ preservation was increased compared to control (**) | None of the tests showed complete recovery to WT results. No other secondary effects were detected |
| (Thomsen *et al.*, 2017) ^58^ | Injection of AAV9-GDNF systemically | Grip strength, disease onset and survival were tested, however only minimal results were observed. | The total number of neurons was increased following treatment, however no difference was seen in the total area | Very little significance; For the behavioural tests, no statistical differences were seen, except for in the forelimb grip strength (*) at 154 days old. Number of neurons was slightly different (*), whereas the total area of the neurons showed insignificant results | Administration of treatment led to slower weight gain, reduction in activity and deficits in working memory. |
| SOD1 KO mice + cells encoding growth factors | | | | | |
| (Islamov *et al.*, 2017) ^59^ | AV vectors expressing VEGF, GDNF and NCAM | Behavioural activity tests show varied results, but mostly an increase following transplantation | Motor neuron survival was not assessed | Mild significance; The comparison between treated and the WT/control mice is significant for horizontal/vertical/exploration activity (*). Survival was increased by a few weeks | No side effects mentioned |
| (Laperle *et al.*, 2023) ^60^ | Induced pluripotent stem cells transduced with GDNFs | Disease onset times and locomotor tests, assessing hindlimb function were not successful | Neuroprotection was enhanced in rats injected with iNPC-GDNF | Mild to no significance; Neuroprotection of retinal photoreceptors were mostly successful (up to ****) and there was mild protection of motor neurons (*). Behavioural tests showed now significance | No side effects shown; Excessive GDNF can cause side effects in patients |
| (Thomsen *et al.*, 2018) ^61^ | Transplanting neural progenitor cells secreting growth factor GDNF | Motor function was improved, shown by testing forelimb and hindlimb function, onset and survival. | Rats that received treatment showed an increase in total number of spinal motor neurons and total neuron area | Mild significance; Behavioural tests showed small significance in the difference between control and treated rats (up to *). For the number of motor neurons, the differences between control and treated was p=0.08, which is generally considered insignificant, however the total area showed higher significance (*) | No adverse effects were observed |
| TDP-43 KO mice + Growth factors | | | | | |
| (Genç et al., 2023) ^62^ | Expression of the active form of HGF | No behavioural studies were performed | HGF delivery helps retain upper motor neurons in the motor cortex | Mild significance; The number of UMNs in the motor cortex shows a significant increase compared to the control (*) and UMN apical dendrite degeneration (a characteristic of ALS) recovered significantly also (****) | They found this method is not toxic, does not increase astrogliosis or microgliosis |
| (Lin *et al.*, 2018) ^63^ | Intramuscular Delivery of scAAV9-hIGF1 | Body weight, rotarod tests and step lengths show improvements compared to controls, as do survival tests and the disease onset times | Number of motor neurons were increased following treatment. The treatment also reduced astroglial and microglial responses in ALS mice | Significant; Weight and rotarod tests show mild significance (*), whereas step length tests show higher (**). Survival tests and disease onsets both have very significant results (P<0.005-0.0001). Number of motor neurons was increased with a significance of P=0.007. | No side effects were mentioned or observed. |
| (Wang *et al.*, 2016) ^64^ | scAAV vectors delivery VEGF | Survival, rotarod, weight and neurological tests showed a slight recovery in function compared to controls | Number of motor neurons were measured and increased slightly following treatment | Mild significance; Behavioural tests show an increase in motor and cognitive function, with a slight significance (ns to *). Motor neuron numbers are increased in comparison to control vectors (*). | No side effects were mentioned or observed. |
| (Wang *et al.*, 2018) ^65^ | Delivery of human IGF1 by scAAV vectors | Weight, time to fall and % survival was tested and showed a recovery of function following treatment | SMI32-positive cells indicated motor neurons – a recovery in numbers was observed compared to controls | Mild significance; behavioural tests show a recovery in function, but the statistical significance is relatively low (ns to *). Motor neuron number show a recovery towards the WT numbers following treatment, and away from the controls (**). | No side effects were mentioned or observed. |
| (Lee *et al.*, 2019) ^66^ | AAV1 vector expressing hepatocyte growth factor (HGF) | Limb strength, latency to fall, body weight and survival tests showed an improvement following treatment | Number of spinal motor neurons (SMNs) increased following treatments compared to the controls | Motor function tests were significant for days 10-20 post-injury with the most significant being day 14. | Cytotoxic effects were not observed |
| (Eleftheriadou *et al.*, 2016) ^67^ | Gene delivery of *IGF-1* using coxsackievirus and adenovirus receptor (αCAR)-targeted vector | Survival, body weight and % affected tests show improvements following treatment. Latency to fall and stride length show similar results | Motor neurons were measured in the lumbar and thoracic spinal cord were increased following αCAR IGF-1 | Significant; Survival was increased by 70 days compared to non-treated mice (P<0.05). Body weight was increased significantly (up to ***) and all behavioural tests show significant improvement (* to ***). Motor neuron numbers were significantly recovered (** and ***) | No side effects were mentioned or observed. |
| Neuromuscular junction and synaptic support | | | | | |
| (Miyoshi *et al.*, 2017) ^68^ | Expression of cytoplasmic protein Dok-7 | Gene therapy enhances survival and motor activity in mice – grip strength and survival tests were used, as well as spontaneous motor activity tests. | Therapy suppresses motor nerve terminal degeneration at the NMJ | Behavioural significance = very low; grip strength and disease onset tests showed no significance. Survival tests showed significance of P<0.01, whereas locomotor tests showed significance of P<0.05. Motor nerve survival showed higher significance (* to ***) | No side effects were shown |
| (Ueta *et al.*, 2020) ^35^ | Expression of cytoplasmic protein Dok-7 | Motor function and muscle strength following treatment was elevated | NMJ innervation was enhanced following therapy | Varied significance; The latency to fall and body weight of the mice showed know significant differences, however rotarod scores improved over time (up to ***). NMJ innervation was significant between control and treatment (up to ***) | Side effects were not tested, still a risk of potential adverse effects |
| (Mancuso *et al.*, 2016) ^69^ | Overexpression of Nrg1-I using AAV | Latency to fall and probability of onset were assessed but no differences were observed | Number of motor neurons was not improved following treatment, neither was the intact axonal number. However NMJs were preserved, as was the amount of neural sprouts. | Low to mild significance; Rotarod test and the probability of disease onset was not improved following treatment. Also, the number of motor neurons and axonal number were similar to the controls. However, the NMJ preservation was improved significantly (**). The number of sprouts found in the neurons were significantly improved as well (**). | No side effects were mentioned or observed. However, the beneficial effect of this treatment was low. |
| (Mòdol-Caballero *et al.*, 2020) ^70^ | Overexpression of neuregulin 1 (NRG1) | Results in a promotion of motor function using locomotion tests | Preserves motor neuron (MN) numbers and decreases glial reactivity | Significant; Rotatod tests were significant (***) against MOCK vectors (tested up to 16 weeks of age). Motor neuron and MNJ preservation was also significant (****). Decreases in astrocytes (**) and microglia (*) reactivity were slightly significant | No side effects mentioned |
| (Mòdol-Caballero, Herrando-Grabulosa, *et al.*, 2021) ^71^ | Overexpression of neuregulin 1 (NRG1) Type I and NRG1 Type III | Functional improvements of SOD1*G93A mice using locomotion tests | Preserves the number of MN and NMJs in effected mice, as well as decreasing astrocyte and microglia reactivity | Mildly significant; Rotarod tests were moderately significant (up to **), with the significance increasing with age of mice. Preservation of MN and NMJs were significant (***) and decreases in astrocyte and microglia reactivity showed small significance (*) | No side effects shown; NRG1-III only showed beneficial effects in female SOD1*G93A mice |
| (Chen *et al.*, 2024) ^72^ | Forced Nuclear receptor interacting-protein (NRIP) expression | Promotion of locomotor activity was tested by tracking their movements in an opaque box, as well as using rotarod and grip force tests | NMJ degeneration and axon terminal denervation at NMJ was reduced and the number of MN and axon potentials were increased | Mild to no significance; Small significance in locomotor improvements was seen (ns or *), with no significant effect seen in rotarod and grip strength tests. Mildly significant effects were seen in NMJ improvements (*) and motor neuron survival (*) | Survival was not improved using this gene therapy at this dose |
| (Nizzardo *et al.*, 2020) ^73^ | Gene therapy expressing *Synaptotagmin 13* (SYT13) | The latency to fall was decreased slightly following treatment and the chance of survival was increased | Motor neuron survival was increased by overexpression of Syt13 | Significant; Inverted grid was only slightly significant, but survival had a higher significance (P= 0.0029). Number of motor neurons and axons was significantly increased following treatment (***) | The number of MN/axons was improved but not fully recovered |
| (Wang *et al.*, 2022) ^74^ | Subpial delivery of neuroprotective *SynCav1* | Distance travelled by rats on wheel was increased following treatment, as was velocity. Body weight, disease onset time and survival was also improved | Value of motor neuron area improved following treatment of *SynCav1*. NMJs were also tested and showed an increase compared to controls | Significant; BW, disease onset and survival were all found to be significantly improved following treatment (*** to ****). Movement assays also showed a mildly significant recovery (* to ***). Motor neurons were also mildly improved (*), whereas the improvement of NMJs was very significant (****). | No toxic effects were found in this study |
| (Mora *et al.*, 2024) ^75^ | Overexpression of Extended Synaptotagmin 1 (*Esyt1)* | Locomotion was improved following therapy - via speed, step frequency, stride length and acceleration tests | Synapses on motor neurons are rescued and survival of motor neurons are increased | Significant; Behavioural tests show significant differences between control and injected (** to ***). Recovery of synapses and motor neuron survival is also significant (* to **) | Side effects were tested for; No observable differences were found in these tests |
| Anti-inflammation/immune modulation | | | | | |
| (Ayers *et al.*, 2015) ^76^ | Comparisons of the efficacy of rAAV2/1, 2/5, 2/8 and 2/9 encoding IL-10 | Survival and body weight tests were used to test phenotype responses. Both tests showed slight recovery, but not much | No quantitative analysis was performed on the amount of motor neurons present | Low significance; Survival assay showed a 15.2% extension to life span following treatment, but there was no difference in the median survival. Body weight analysis showed no significant changes from control to treated mice. | No side effects were mentioned or observed. |
| (Rossi *et al.*, 2018) ^77^ | CNS-delivery of intereukin-4  (IL-4) | Body weight, survival and latency to fall were all improved following treatment | Alternative activation of microglia is enhanced by IL-4 expression | Mild significance; Behavioural tests show an improvement with some significance (up to **). The significance of microglia enhancement varies on the test (* to ***) | IL-4 did not improve motor neuron cell survival |
| (Leyton-Jaimes, Kahn and Israelson, 2019) ^78^ | AAV vectors to overexpress macrophage migration inhibitory factor (MIF) | Survival tests showed a slight recovery following treatment, similar with disease onset and progression. | No neuroprotective tests were performed. | Mild significance; Survival was increased in SOD1*G93A mice (**) as well as loxSOD1*G37R mice (**). Disease onset had similar results (*). Progression was only tested in SOD1*G93A mice, but the difference was again significant (**) | Drug is shown to be safe and well-tolerated with no adverse effects |
| Glutamate regulation and excitotoxicity control | | | | | |
| (Li *et al.*, 2015) ^79^ | Overexpression of glutamate transporter, GLT1 | Group strength, survival and disease onset was not affected by treatment | Motor neuron numbers were also not affected by GLT1 overexpression | Insignificant; No changes were found between controls and an overexpression of GLT1 | This treatment was identified as an ineffective therapy for ALS |
| (Eykens *et al.*, 2021) ^80^ | Delivery of monocarboxylate transporter 1 (MCT1) to oligodendrocytes | No behavioural tests were performed | No difference between number of motor neurons between the treated and controls | No significance; Survival of mice was not improved at all between treated and control. No significance was found in any other assays | No side effects mentioned |
| (Wang *et al.*, 2017) ^81^ | Overexpression of D-amino acid oxidase (DAO) | Survival, weight and latency to fall was measured, and showed a moderate recovery following treatment | Treatment alleviated motor neuron loss, axon degeneration and muscle atrophy | Mild significance; In female mice the survival was extended by 8 days but had no effect in males. Behavioural assays showed a range of results (from ns to *). MN and axon numbers were increased by a small significance (*) | DAO selectively extended survival in female mice; No effect in body weight for either gender |
| (Benkler *et al.*, 2016) ^82^ | Lentivirus encoding: EAAT2, GDH2 and NRF2 | Neurological tests showed a synergistic effect with each gene. Locomotor tests showed the same | The number of motor neurons in increased for individual treatments, but the mix had the greatest effect | Mild significance; Behavioural tests show significance (* to **) when analysed, however some tests did not show statistics. Motor neuron numbers were significant (P<0.001) between each step up (individual -> synergy) | No side effects were observed/ tested |
| (Ohashi *et al.*, 2021) ^83^ | Bone marrow-derived cells infecting with LVs expressing GLT1 | Rotarod and survival tests were improved following treatment compared to controls | Motor neurons were preserved in treated mice compared to the controls | Mild significance; For behavioural tests, rotarod results showed significance (*), as did the survival assay (*). Motor neuron number was significantly higher following treatment (*) | No side effects mentioned |
| Cellular stress response | | | | | |
| (Valenzuela *et al.*, 2025) ^84^ | Expression of transcription factor XBP1 | Disease onset was delayed and lifespan was extended. Hanging tests and body weight analysis also showed positive results | Neuroprotection was not analysed in this study | Mildly significant; Behavioural tests show some significance between the therapy and Mock vectors (P<0.05) | Male mice did not have beneficial effects from the therapy |
| (Jackson *et al.*, 2015) ^85^ | AAV9 vector used to express mycUPF-1 in rat spinal cords | Many behavioural tests were performed, including rotarod, locomotor activity and rearing, showing an improvement following treatment | No motor neuron number tests were performed. | Significant; Behavioural tests show a significant recovery following treatment of TDP-43 expression (P<0.05) | No differences were detected while testing for adverse effects |
| (Wen *et al.*, 2024) ^86^ | Increased expression of Optineurin (OPTN) | Muscle weakness was improved following therapy, as well as survival. Stride length, rotarod and weight tests also showed improvement | The number of MN was increased following therapy | Mild significance; Small significance seen in behavioural tests: Stride length and rotarod (**) and weight tests (*). Survival showed significance of P<0.01. Mild significance of motor neuron survival was also seen (**) | Increase in LC3-II and a decrease in p62 indicate enhancement of autophagic processes |
| (Amado *et al.*, 2025) ^87^ | AAV delivery of RNAi targeting ataxin-2 | Survival, latency to fall and climbing tests showed a recovery of function following treatment | Lower motor neuron numbers were measured and showed similar results to WT | Significant; Behavioural tests show significant results (** to ****), with the climbing tests showing higher results than even the WT controls (** to ****). A recovery of MN number is shown (all ns compared to WT); however the controls show no significance for most as well. | No adverse effects were found. However, for MN number test, the controls didn’t have the effect they should have. |

**Supplementary Table 3.** Numerical data used for meta-analysis.

|  | **Motor neuron no.** | | | | **Mean AnimalSurvival** | | | **Rotarod (Latency to fall) (s)** | | |
| --- | --- | --- | --- | --- | --- | --- | --- | --- | --- | --- |
| **Study ID** | Controls | Treatment | P value | N numbers | Controls | Treatment | P value | Controls | Treatment | P value |
| **SOD1 KO mice** | | | | | | | | | | |
| **(Rochat *et al.*, 2022) ^36^** | WT mice = 25 +/- 1.1 SOD1 mice + no treatment = 10 +/- 0.9 | SOD1 mice - miR SOD1 = 14.6 +/- 0.9 | **p < 0.01 between treated and untreated mice | WT - N = 5 Untreated mice - N = 6  miR SOD1 treatment - N = 6 | NA | NA | NA | No numerical data | No numerical data | up to **p<0.01 |
| **(Y. A. Chen *et al.*, 2023) ^37^** | WT, LacZ injected mice = 25.6 +/- 3.7  SOD1, LacZ injected mice = 16 +/- 3.3 | SOD1, sgSOD1 injected mice = 27.7 +/- 3.7 | ns between WT and treated mice  ***p< 0.001 between WT and SOD1 LacZ injected mice Full recovery observed | WT - N = 5 LacZ injected - N = 7 sgSOD1 mice - N = 5 | NA | NA | ****p<0.0001 | No numerical data | No numerical data | No numerical data |
| **(Lim *et al.*, 2020) ^38^** | MRosa26 as control vector = 3.49 +/- 0.9 | hSOD1 treatment = 4.43 +/- 0.74 | *p < 0.05 | n = 7 per group | MRosa26 as control vector = 127 +/- 7.2 days | hSOD1 treatment = 139.4 +/- 10.8 days | ***p < 0.001 | No numerical data | No numerical data | *p<0.05 |
| **(Biferi et al., 2017) ^39^** | WT = 18.4 +/- 0.4 Non-injected mice = 9.8 +/- 0.15 Control injections = 8,8 +/- 0.15 | hSOD1 treated mice = 12.1 +/- 0.3 | ****p < 0.0001  between treated and untreated + treated and control | n = 6, three sections per mouse | Non-injected SOD1 mice = 112.8 days +/- 0.9 days | AAV10-hSOD1 = 195 days +/- 6.7 days | ****p<0.0001 | WT mice = 59.2 +/- 2.6  Non-infected transgenic mice = 21.4 +/- 2.2 | hSOD1 treated mice = 31.0 +/- 3.3 | *p<0.05 between treated and untreated mice. ****p<0.0001 between treated and WT |
| **(Dirren et al., 2015) ^40^** | WT PBS = a) 44.7 +/- 1.9, b) 33.3 +/- 0.7 Transgenic mice with PBS = a) 21 +/- 1.7 , b) 15.2 +/- 1 | Injection of miR SOD1 = a) 26.9 +/- 0.8, b) 25.5 +/- 1.4 | between transgenic PBS mice and treated mice = a) p= 0.06, b) ***p< 0.001 | WT - N = 9 Transgenic PBS - N = 8 miR SOD1 injection - N = 6 | No numerical data | No numerical data | ns | WT PBS = 193.2 +/- 16.1 Transgenic PBS = 30.5 +/- 8.5 | Injection of miR SOD1 = 83 +/- 14.6 | *p<0.05 between treated and untreated mice. ***p<0.001 between treated and WT |
| **(S. K. Chen *et al.*, 2024) ^41^** | NA | NA | NA | NA | NA | NA | NA | NA | NA | NA |
| **(Stoica *et al.*, 2016) ^42^** | untreated SOD1 mice = 6 +/- 1 NTG mice = 18.4 +/- 2.2 | amiRSOD1 treated mice = 12 +/- 1.6 | **p< 0.001 between treated and untreated SOD1 mice | Not stated | Untreated mice = 137 days | amiR-SOD1 treatment = 206 days | ****p<0.00001 | NA | NA | NA |
| **(Duan et al., 2020) ^43^** | LacZ injected mice = 1.7 +/- 0.2 | sg5-treated mice = 13.3 +/- 0.6 | ***p < 0.001 | N = 12-14 | AAV9-sgLacZ = 130 +/- 1.2 days | AAV9-sgRNA5 = 201 +/- 12.1 days | ***p<0.001 | No numerical data | No numerical data | ****p<0.0001 |
| **(Gaj *et al.*, 2017) ^44^** | untreated SOD1 mice = a) 4.4 +/- 1.3, b) 4 +/- 0.8 | saCas9-hSOD1 mice = a) 11.7 +/- 5.3, b) 9.3 +/- 2 | a) *P<0.05  b) ***P<0.001 | n = 4 per group | Untreated mice = 125 +/- 3.5 days EGFP injected mice = 124 +/- 2.8 days mRosa26 injected controls = 122.8+/- 6.1 days | hSOD1 treatment = 152.4 +/- 7.9 days | ***p<0.0005 between hSOD1 and EGFP injected + hSOD1 and mRosa26 injected mice + hSOD1 and untreated mice | No numerical data | No numerical data | from **p<0.01 to ***p<0.001 |
| **(Borel et al., 2016) ^45^** | NA | NA | NA | NA | GFP injected control = a) 108 days, b) 131 days | miRSOD1 injected mice = a) 130 days, b) 158 days No standard deviation mentioned | a) *p<0.05 b) ****p<0.0001 | No numerical data | No numerical data | **p<0.01 |
| **(Keeler *et al.*, 2020) ^46^** | NTG mice = 30.8 +/- 1.5  Untreated SOD1 mice = 14.8 +/- 2.2 | AAV-miR(SOD1) = 15.9 +/- 3.5 | ns - there appears to be no difference from treatment | NTG mice - N = 6 Untreated SOD1 mice - N = 3 miRSOD1 mice - N = 4 | Non-injected SOD1 mice = 137.5 days | AAV-miRSOD1 mice = 187 days | ****p<0.0001 | NA | NA | NA |
| **(Li *et al.*, 2017) ^47^** | Non-transgenic mice = 14.7 +/- 0.7  PBS injected mice = 5.6 +/- 0.6 | amiRSOD1 treated mice = 9 +/- 0.5 | **p<0.01  between treated and PBS-treated, as well as treated and non-transgenic mice | n = 3 animals and 3 sections were quantified from each animal | PBS injected mice = a) 128.8 = +/- 12.8 days, b) 132.7 +/- 10.5 days | Slow injected SOD1 treatment = a) 139.7 +/- 8.7 days, b) 144.7 +/- 8.9 days Fast inject treatment = a) 134+/- 8.4 days, b) 141.4 +/- 11.1 days | p<0.05 | NA | NA | NA |
| **(Lind *et al.*, 2020) ^48^** | NA | NA | NA | NA | Vehicle injection: a) 129 +/- 1 day, b) 134 +/-3 days | miRSOD1 injection: a) 127 +/- 4 days, b) 141 +/- 2 days | p<0.05 for b (females) but p>0.05 for a (males) | NA | NA | NA |
| **(Iannitti *et al.*, 2018)** ^49^ | Scrambled control vector = 1.75 +/- 1.2 | scAAV9-hSOD1 treatment = 6.75 +/- 1.6 | *p<0.05 | n = 10 sections/mouse, 3 mice per treatment | Scrambled control vector = 138 +/- 1.9 days | scAAV9-hSOD1 = 157 +/ 2.9 days | ****p<0.00001 | Scrambled control vector = 14.4 +/- 4.2 | scAAV9-hSOD1 = 88.1 +/- 16.1 | ***p<0.001 |
| **SOD1 in combination** | | | | | | | | | | |
| **(Baird et al., 2024) ^50^** | Untreated mice = 5.59 +/- 0.9 | AAV9-Gal1 = 3.2 +/- 0.4 AAV9-SOD1-shRNA = 12.9 +/-0.7 AAV9-SOD1-shRNA-Gal1 treatment = 11.5 +/- 0.7 | ****p < 0.0001  between treated and untreated | n = 3 per group, 12 sections per sample | Untreated mice = 139 days +/- 17.8 days | Gal1 mice = 138 days +/- 17.7 days SOD1 mice = 190 days +/-37.5 days Gal1+SOD1 mice = 228 days +/- 39 days | ****p<0.0001 between combination and untreated mice + between untreated and SOD1 + between SOD1 and combination | No numerical data | No numerical data | No numerical data |
| **(Frakes *et al.*, 2017) ^51^** | NA | NA | NA | NA | Non-injected SOD1 mice = 136.9 days +/-0.9 days | Cre-injected mice = 157.9 days +/- 1.8 days  shRNA SOD1 injected = 177.4 days +/- 1.9 days Combination = 189.1 days +/- 2.7 days | All treatment is significant against control: ****p<0.0001 Combination treatment is significantly better than all | No numerical data | No numerical data | No data for significance |
| **TDP-43** | | | | | | | | | | |
| **(Peethambaran Mallika *et al.*, 2025)** ^52^ | WT mice with treatment an GFP = 21.2 +/- 2 TDP-43 KO with GFP = 10 +/- 2.4 | TDP-43 KO with treatment = 16.2 +/- 3 | ****p < 0.0001  between treatment and GFP | n = 8 per group | No numerical data | No numerical data | No numerical data | NA | NA | NA |
| **(Pozzi *et al.*, 2019)** ^53^ | NA | NA | NA | NA | No numerical data | No numerical data | *p=0.03 | NA | NA | NA |
| **(Wu *et al.*, 2025)** ^54^ | RPS + GFP = 2180 +/- 70 per mm2 AAV8+scramble = 90 +/- 20 per mm2 | AAV8-TDP43 = 1700 +/- 100 per mm2 | ****p < 0.0001  between treatment and scramble | GFP-injected - N = 6  Scramble-injecte d- N = 5 TDP-43 injected - N = 6 | NA | NA | NA | WT PBS = 300 scramble vector = 55 | siR-TDP-43 treatment = 130 +/- 20 | *p< 0.05 |
| **SOD1 KO mice + AAV encoding Neurotrophic Factors** | | | | | | | | | | |
| **(Le *et al.*, 2025)** ^55^ | Control virus = 2.6 +/- 0.4 WT mice = 8.5 +/- 1 | BDNF-injected = 4.3 +/- 0.6 GAS6 injected = 5.3 +/- 0.15 | **p<0.01 between control virus and BDNF injected ****p<0.0001 between control virus and Gas6 injected | n = 3-4 per group, mixed sexes | NA | NA | no statistical significance was shown between control and any treatment | No numerical data | No numerical data | ns difference |
| **(Gross *et al.*, 2020)** ^56^ | GFP injected mice = 1.72 +/- 0.74 Formulation buffer = 2.72 +/- 1.05 WT = 6.8 | Injected mice = 5.84 +/- 1.51 | ****p<0.0001 between treated and FB-treated mice  ns between treated and WT | AAV2-GFP - N = 3 Formulation buffer - N = 13 WT - N = 7 Injeected mice - N = 13 | Formulation buffer = 125.1 +/- 8.25 days | AAV2-NRTN - dose = 2.6 x 10^10 vg/mouse  129.9 +/- 9.61 days | ns in overall survival | NA | NA | NA |
| **(Mòdol-Caballero, García-Lareu, *et al.*, 2021)** ^57^ | WT mock = 21.4 +/- 1.5 WT GDNF = 21.4 +/- 1 Transgenic mock = 9.1 +/- 0.6 | SOD1 GDNF = 16.6 +/- 1 | *** p<0.001 between treatment and transgenic mock | NA | NA | NA | NA | No numerical data | No numerical data | up to *p<0.05 |
| **(Thomsen *et al.*, 2017)** ^58^ | AAV9(-) injected mice : a) 9.7 +/- 0.7, b) 0.28 +/- 0.06 | AAV9-GDNF injected mice: a) 12.3 +/- 0.8, b) 1.5 +/- 0.3 | *p <0.05 - for comparisons between both A's and B's | n = 4 per group | No numerical data | No numerical data | ns in overall survival | NA | NA | NA |
| **SOD1 KO mice + cells expressing neurotrophic factors** | | | | | | | | | | |
| **(Islamov *et al.*, 2017)** ^59^ | NA | NA | NA | NA | NA | NA | NA | NA | NA | NA |
| **(Laperle *et al.*, 2023)** ^60^ | Untreated = 1.1 +/- 0.27 | iNPC-GDNF treated rats = 1.7 +/- 0.8 | *p < 0.05 | Untreated - N = 7  iNPC-GDNF treated - N = 10 | NA | NA | ns difference between treated and untreated | NA | NA | NA |
| **(Thomsen *et al.*, 2018)** ^61^ | Non-injected SOD1 mice = 6.7 +/- 0.7 | hNPC*GDNF treated mice = 7.6 +/- 0.6 | ns - p=0.08 | 8 sections counted total - not stated how many rats per group | Non-injected rats = 170 days | hNPC*GDNF receiving rats = 184 days | *p<0.05 | NA | NA | NA |
| **TDP-43 KO mice + Growth factors** | | | | | | | | | | |
| **(Genç *et al.*, 2023)** ^62^ | Non-transgenic mice = 77.3 +/- 6.7  MCS-injected control mice = 34.7 +/- 2.3 | HGF-injected mice = 52 +/- 2.7 | *p<0.05 | n ≥ 3 biological replicates | NA | NA | NA | NA | NA | NA |
| **(Lin *et al.*, 2018)** ^63^ | AAV9-GFP = 4.69 +/- 0.9 | AAV9-hIGF1 = 9.9 +/- 0.5 | ** p = 0.007 | Not stated | NA | NA | ****p<0.0001  (except for males injected at 90 days -> p=0.0082) | No numerical data | No numerical data | *p<0.05 |
| **(Wang *et al.*, 2016)** ^64^ | AAV9-GFP = 6.1 +/- 1.3 | AAV9-VEGF = 8.8 +/- 1.9 | *p<0.05 | n = 15 per group | AAV9-GFP = a) 131 days, b) 129 days | AAV9-VEGF = a) 143 days, b) 142 days | a) **p=0.001 b) **p = 0.001 | No numerical data | No numerical data | low significance |
| **(Wang *et al.*, 2018)** ^65^ | Non-transgenic mice = 13.1 +/- 1 GFp-injected mice = 5.7 +/- 0.7 | IGF1-injected mice = 8.1 +/- 0.8 | **p<0.01 between IGF1- and GFP-treated mice | n = 3 per group | AAV9-GFP = 130 +/- 3.9 days | AAV9-IGF1 = 140 +/- 3.2 days | *p<0.05 | No numerical data | No numerical data | up to *p<0.05 |
| **(Lee *et al.*, 2019)** ^66^ | Non-transgenic mice = 35.6 +/- 3.75  Control vector = 17.5 +/- 0.9 | rAAV1-HGF vector = 41.2 +/- 2.8 | *p<0.05 between treated and non-treated transgenic mice | Not stated | Control vector = 145.5 days | HGF injected vector = 155 days | *p = 0.0115 | No numerical data | No numerical data | ns difference |
| **(Eleftheriadou *et al.*, 2016)** ^67^ | Non-treated SOD1 mice = a)11.9 +/- 0.85, b) 10 +/- 1.3 VSVG IGF1 mice = a) 6.8 +/- 0.2, b) 6.8 +/- 0.85 | aCAR IGF1 treatment = a) 18.7 +/- 1.7, b) 17.5 +/- 1.4 | between non-treated and treated = **p<0.01 between VSVG IGF1 and aCAR IGF treatment = ***p<0.001 | n = 8 per group | notreated control mice = 155 days VSVG IGF1 mice = 160 days | aCar IGF 1 treatment = 169 days | ****p<0.0001 between aCar and nontreated control *p=0.012 between aCar and VSVG IGF1 treated | No numerical data | No numerical data | from *p<0.05 to ***p<0.001 |
| **Neuromuscular junction and synaptic support** | | | | | | | | | | |
| **(Miyoshi *et al.*, 2017)** ^68^ | NA | NA | NA | NA | Untreated mice = 50.3 +/- 3.1 days | AAV-D7-treated mice = 64.2 +- 3.3 days | **P = 0.0023 | NA | NA | NA |
| **(Ueta *et al.*, 2020)** ^35^ | NA | NA | NA | NA | NA | NA | NA | No numerical data | No numerical data | up to ***p<0.001 |
| **(Mancuso *et al.*, 2016)** ^69^ | WT = 336.7 +/- 3 SOD1 non-transgenic = 150 +/- 10 | Transgenic Nrg1-I = 170 +/- 16.7 | ns increase between transgenic and non-transgenic | n = 10 animals per group, balanced sexes | NA | NA | NA | No numerical data | No numerical data | ns difference |
| **(Mòdol-Caballero *et al.*, 2020)** ^70^ | WT = 20.7 +/- 1.1 SOD Mock = 5.9 +/- 0.5 | SOD NRG1-I = 12.4 +/- 0.5 | ****p < 0.0001  between treatment and SOD Mock | WT - N = 3 SOD Mock - N = 3-4 SOD NRG1-I = 7 | NA | NA | NA | No numerical data | No numerical data | up to ***p<0.001 |
| **(Mòdol-Caballero, Herrando-Grabulosa, *et al.*, 2021)** ^71^ | WT = 20.1 +/- 1 SOD Mock = 7.4 +/- 0.7 | SOD NRG1-I + NRG 1-III = 12 +/- 1.1 | ***p < 0.001  between treatment and SOD Mock | WT - N = 6-7  SOD Mock - N = 8  SOD NRG1-I + NRG1-III = 6 | NA | NA | NA | No numerical data | No numerical data | up to **p<0.01 |
| **(Chen et al., 2024)** ^72^ | WT = 25 +/- 0.9  AAV GFP = 14.91 +/- 0.98 | AAV-NRIP = 17.81 +/- 0.7 | *p = 0.0302 between treatment and AAV GFP | WT - N = 9  AAV-GFP - N = 11 AAV-NRIP - N = 14 | NA | NA | no significant difference | AAV-GFP = 106.7 +/- 26.7 | AAV-NRIP = 113.3 +/- 18.3 | ns difference |
| **(Nizzardo *et al.*, 2020)** ^73^ | WT = 22.8 +/- 0.4 AAV9-null = 10 +/- 0.6 | AAV9-syt13 = 16.4 +/- 0.4 | ***p < 0.0001 between treated and null mice | n = 30 slices counted/group n = 3 mice/group | AAV9-null = 140 +/- 5 days | AAV9-Syt13 = 160 +/- 14 days | **p=0.0029 | NA | NA | NA |
| **(Wang *et al.*, 2022)** ^74^ | WT = 15.6 +/- 1.25 Untreated SOD1 mice = 6 +/- 0.6 Control vector SOD1 mice = 5 +/- 0.2 | SynCav1 treated mice = 11.8 +/- 1.1 | *p < 0.05 between Syncav1 treated mice and control mice | WT - N = 4 Untreated - N = 3 Control vector - N = 3 Treated mice - N = 5 | Naïve transgenic mice = a) 159.5 days, b) 162.5 days Control mice = a) 162 days, b) 165 days | SynCav1 treated mice = a)178 days, b) 181 days | a) **p=0.0046 b) ****p<0.0001 | NA | NA | NA |
| **(Mora *et al.*, 2024)** ^75^ | SOD1 mice + no treatment = 6.6 +/- 0.8 | SOD1 + EN1cre treatment = 10.9 +/- 0.9 | ** P = 0.0074 | 10 hemicords used per group No treatment - N = 5 EN1cre treatment - N = 4 | SOD1 mice + no treatment = 162.7 +/- 6 days | SOD1 + EN1cre treatment = 156.5 +/- 9,6 days | ns in overall survival | NA | NA | NA |
| **Anti-inflammation/immune modulation** | | | | | | | | | | |
| **(Ayers et al., 2015)** ^76^ | NA | NA | NA | NA | Control mice = 119 days EGFP mice = 112 days | IL-10 infected mice = 133 No standard deviation mentioned | **p<0.01 | NA | NA | NA |
| **(Rossi *et al.*, 2018)** ^77^ | WT = 10.5 +/- 0.24 GFP-treated = 6.5 +/- 0.5 | IL-4 treated mice = 5.8 +/- 0.4 | ns | n = 3 for each group | G93A-GFP-LV controls = 131 days | G93a-IL-4-LV = 129 days | ns in overall survival | No numerical data | No numerical data | up to **p<0.01 |
| **(Leyton-Jaimes, Kahn and Israelson, 2019)** ^78^ | NA | NA | NA | NA | AAV2/9-GFP = 148 +/- 6 days | AAV2/9-MIF = 178 +/- 7 days | **p<0.01 | NA | NA | NA |
| **Glutamate regulation and excitotoxicity control** | | | | | | | | | | |
| **(Li *et al.*, 2015)** ^79^ | GFP injected mice = 17.6 +/- 1.8 | GLT1 injected mice = 16.5 +/- 1.1 | ns - there appears to be no difference from treatment | n = 7-8 per group | NA | NA | ns difference between treated and GFP treated | NA | NA | NA |
| **(Eykens *et al.*, 2021)** ^80^ | Empty = 2.9 +/- 0.75   GFP-transduced mice = 3.3 +/- 0.8 | MCT1-transduced mice = 3.3 +/- 0.7 | ns - there appears to be no difference from treatment | n = 4-5 mice per group | Empty = 154.5 days GFP = 156 days | MCT1-transduced = 155 days | ns shown on survival | NA | NA | NA |
| **(Wang *et al.*, 2017)** ^81^ | AAV9-GFP = 5.5 +/- 1.3 | AAV9-DAO = 8.3 +/-1.3 | *p<0.05 | n = 4 per group | AAV9-GFP = 130 +/- 4.1 days | AAV-DAO= 136 +/- 2.2 days | **p=0.005 | No numerical data | No numerical data | very low significance |
| **(Benkler et al., 2016)** ^82^ | GFP-injected SOD1 mice = 4.6 +/- 0.2 Saline-treated mice = 3.75 +/- 0.2 | Mixed treatment (EAAT2/NRF2/GDH2) = 6.8 +/- 0.3 | **p < 0.01 | n = 4 animals and 12-14 spinal cord splices | Saline-infected mice = a) 148 days +/- 4.5 days, b) 159 days +/- 4.5 days GFP-infected mice = a) 153 days +/- 2.5 days, b) 159 days +/- 3 days | Mixed treatment (EAAT2/NRF2/GDH2) = a) 166 days +/- 2.5 days, b) 180 days +/-2.5 days | ***p<0.001 | Saline-infected mice = a) 116.6 +/- 4.4, b) 126.5 +/- 4.2 GFP-infected mice = a)123.7 +/- 2.4, b) 127.6 +/- 2.4 | Mixed treatment = a) 133.8 +/- 1.1, b) 158.5 +/- 1.6 | ***p<0.001 |
| **(Ohashi *et al.*, 2021)** ^83^ | Untreated = 1 +/- 0.08 YFP-treated = 0.92 +/- 0.06 | GLT1 treated mice = 1.96 +/- 0.3 | **p< 0.01 between treated groups and the other groups | n = 5 in each group | No numerical data | No numerical data | No numerical data | No numerical data | No numerical data | up to *p<0.05 |
| **Cellular stress response** | | | | | | | | | | |
| **(Valenzuela *et al.*, 2025)** ^84^ | NA | NA | NA | NA | No numerical data | No numerical data | **P=0.003 | No numerical data | No numerical data | up to **p<0.01 |
| **(Jackson *et al.*, 2015)** ^85^ | NA | NA | NA | NA | NA | NA | NA | No numerical data | No numerical data | *p<0.05 |
| **(Wen *et al.*, 2024)** ^86^ | AAV9-GFP = 4.3 +/- 0.2 | AAV9-OPTN = 7.1 +/- 0.2 | **p < 0.01 | n = 3 mice, 6 splices per mouse | AAV-GFP = 141.5 days | AAV-OPTN = 128 days | **p<0.01 | No numerical data | No numerical data | up to **p<0.01 |
| **(Amado et al., 2025)** ^87^ | WT = 19 +/- 4 SOD1 non-transfected mice = 8.5 +/- 3.5 | Transgenic miATXN2 mice = 23 +/- 8 | *p<0.05 between treated and non-treated mice | N = 4-6 mice/group at each level, 4-10 sections/mouse per level | Buffer-treated mice = 22 days +/- 1 day | miATXN2 treated mice = 33.5 days +/- 3 days | ***p<0.0005 | WT mice = 231.5 +/- 12.5  Transgenic mice + buffer = 39.5 +/- 11.8 | miATXN2 treatment = 110.5 +/- 15 .8 | **p<0.01 between buffer and miATXN2 treatment, ****p<0.0001 between WT and miATXN2 mice |

A

B

**Supplementary Figure 1.** Forest plots to demonstrate results of meta analysis stratified by delivery method. Forest plots show changes in ChAT^+^ motor neurons when delivered by (**A**) ICV and (**B**) IM.

**Supplementary Figure 2.** Forest plots to demonstrate results of meta analysis stratified by delivery method. Forest plots show changes in NeuN^+^ motor neurons when delivered by IT.
